# Layered double hydroxide-seaweed composites promote seed germination and seedling growth: a new generation of biostimulants

**DOI:** 10.3389/fpls.2025.1681803

**Published:** 2025-10-16

**Authors:** Adrian Alejandro Espinosa-Antón, Rosalba Mireya Hernández-Herrera, Carla Vanessa Sánchez-Hernández, Gregorio Guadalupe Carbajal-Arízaga, Fabián Alejandro Rodríguez-Zaragoza

**Affiliations:** ^1^ Doctorado en Cs. en Biosistemática, Ecología y Manejo de Recursos Naturales y Agrícolas, Centro Universitario de Ciencias Biológicas y Agropecuarias, Universidad de Guadalajara, Zapopan, Mexico; ^2^ Departamento de Botánica y Zoología, Centro Universitario de Ciencias Biológicas y Agropecuarias, Universidad de Guadalajara, Zapopan, Mexico; ^3^ Departamento de Producción Agrícola, Centro Universitario de Ciencias Biológicas y Agropecuarias, Universidad de Guadalajara, Zapopan, Mexico; ^4^ Departamento de Química, Centro Universitario de Ciencias Exactas e Ingenierías, Universidad de Guadalajara, Guadalajara, Mexico; ^5^ Departamento de Ecología Aplicada, Centro Universitario de Ciencias Biológicas y Agropecuarias, Universidad de Guadalajara, Zapopan, Mexico

**Keywords:** alkaline seaweed extracts, layered double hydroxides, seed vigor, growth promotion, rooting activity

## Abstract

**Introduction:**

Agricultural producers worldwide face increasing pressure to ensure food security while contending with the adverse effects of climate change and unsustainable farming practices. Layered double hydroxides (LDHs) have been applied in agriculture due to their versatile properties as slow-release and efficient carriers, while seaweed extracts are widely used as plant biostimulants. However, their integrated use as nanostructured composites for enhancing seed germination and seedling growth remains largely unexplored.

**Methods:**

In this study, we addressed this gap by synthesizing composites that combine cationic LDH nanosheets with anionic compounds present in alkaline extracts of the brown seaweed *Sargassum liebmannii* (LDH-Sargassum) and the green seaweed *Ulva ohnoi* (LDH-Ulva). The physicochemical properties of the LDH-seaweed composites were characterized using X-ray diffraction (XRD), thermogravimetric analysis (TGA), differential scanning calorimetry (DSC), Fourier-transform infrared spectroscopy (FT-IR), dynamic light scattering (DLS), scanning electron microscopy (SEM), and energy-dispersive X-ray spectroscopy (EDS). The biological efficacy of the composites was evaluated through seed germination and early seedling development assays in tomato (*Solanum lycopersicum* L. cv. Rio Grande) and root induction assays in mung bean (*Vigna radiata* L.) cuttings.

**Results:**

Physicochemical analyses confirmed that approximately 12% of the composite mass corresponded to seaweed-derived molecules adsorbed onto the LDH surface. Both LDH-Sargassum and LDH-Ulva significantly improved germination and seedling growth at lower concentrations (1.57, 3.14, and 6.28 mg·mL⁻¹), while higher doses (12.56–50.25 mg·mL⁻¹) produced effects that were either comparable to those of the control or slightly better. Notably, both composites enhanced root architecture in mung bean cuttings by increasing root number, length, and dry weight. These results highlight the potential of LDH-seaweed composites as effective biostimulants, particularly in promoting early-stage root development by improving root branching, size, and biomass. Importantly, LDH-Sargassum at 6.28 mg·mL⁻¹ emerged as a promising natural alternative to synthetic root-promoting agents.

**Discussion:**

This study demonstrates, for the first time, the feasibility of LDH-seaweed composites as next-generation nanostructured phycobiostimulants, providing insights into their plant interactions and identifying optimal application dosages. Overall, these findings provide a foundation for implementing LDH-seaweed composites as a sustainable strategy to reduce agrochemical inputs and advance towards food security through bio-based nanotechnologies.

## 1 Introduction

The agricultural sector is facing ever-increasing global challenges in meeting production demands, including population growth, the degradation of natural resources, and the escalating impacts of climate change (Shukla et al., 2025). Although the use of synthetic fertilizers and agrochemicals has notably contributed to global food production, their indiscriminate and often excessive application has resulted in adverse consequences for environmental health, biodiversity, soil fertility, and human well-being (Singha Roy et al., 2022; Shukla et al., 2025). While agrochemicals are often necessary to ensure food security, minimizing their environmental impact is critically important. Indeed, there is an urgent need to develop more sustainable and innovative agricultural practices that are able to meet the increasing demand for food while minimizing environmental harm (Ali et al., 2021; Zhang et al., 2025).

In this context, nanobiotechnology has emerged as a promising platform to enhance agricultural productivity, improve plant resilience to stress, and support environmental sustainability (Vithanage et al., 2017; Peng and Qin, 2024). To date, various nanomaterials have been investigated for this purpose, with layered double hydroxides (LDHs), also known as hydrotalcite-like compounds or anionic clays, proving to be particularly noteworthy and promising due to their tunable structure, biocompatibility, and multifunctionality (de Castro et al., 2020; Kumari et al., 2023; Ahmad et al., 2024).

LDHs are two-dimensional lamellar nanostructures composed of positively charged metal hydroxide layers with a brucite-like configuration [Mg(OH)_2_]; the partial substitution of divalent cations (e.g., Mg²^+^) by trivalent cations (e.g., Al³^+^ and Fe³^+^) generates excess positive charge, which is balanced by interlayer anions and water molecules (Xu et al., 2006; Matsuda et al., 2023). Notably, LDHs can accommodate a wide variety of inorganic and organic anions through surface adsorption or intercalation in the interlayer space (Kumari et al., 2023; Kovalenko et al., 2024). In addition, their chemical and thermal stability, pH-responsive solubility, null or low toxicity, and capacity for controlled release make LDHs prime candidates for agricultural applications (Singha Roy et al., 2022; Torbati et al., 2024). Specially, their ease of preparation and low production cost enable large-scale manufacturing, thereby facilitating their use in agricultural systems (Zhang et al., 2025).

Recent studies have demonstrated the potential of LDH-based materials to act as slow-release carriers for fertilizers, herbicides, pesticides, and plant growth regulators, as well as effective adsorbents for environmental contaminants in soil and water (Shlar and Poverenov, 2021; Singha Roy et al., 2022; Kovalenko et al., 2024). It has been documented that the foliar application of LDH nanoparticles to enhance nutrient uptake, improve photosynthetic efficiency, stimulate plant growth and productivity, and trigger both primary and secondary metabolic pathways (Hashem et al., 2024; Wang et al., 2024). Furthermore, their use as nanoplatforms for the delivery of nucleic acids and bioactive molecules into plant systems has opened new avenues to improve crop production and sustainable farming practices (Wu et al., 2022; Komarova et al., 2023). Thus, the incorporation of functional biomolecules into the structure of LDHs offers a viable strategy for their targeted delivery and enhanced bioavailability in plant-soil systems.

In contrast to the use of LDHs, seaweed extracts have long been used in agriculture and horticulture as natural biostimulants and have been recognized for their ability to improve seed germination, seedling vigor, and crop productivity (Craigie, 2011; Shukla et al., 2025). These natural extracts are complex mixtures containing polysaccharides, amino acids, phytohormones, minerals, and secondary metabolites, whose biological effects are highly dependent on the seaweed species, the extraction method, and the applied concentration (Godlewska et al., 2017; Mzibra et al., 2018; Santos et al., 2023). Although maximum benefits are achieved at optimal doses, excessive application may induce phytotoxicity or growth inhibition, underscoring the need for a careful evaluation of the dose-response relationship when developing novel seaweed extract-based formulations (Hernández-Herrera et al., 2014; Ghaderiardakani et al., 2019; Di Filippo-Herrera et al., 2021).

Brown and green seaweeds, particularly *Sargassum liebmanii* J. Agardh and *Ulva ohnoi* M. Hiraoka & S. Shimada, have become important raw materials to produce bioactive extracts for agricultural use, given that they are naturally abundant and rich in bioactive compounds (Hernández-Herrera et al., 2014; Prisa and Spagnuolo, 2022; ZarraonaIndia et al., 2023). Indeed, the cell wall polysaccharides of *Sargassum* (alginate) and *Ulva* (ulvan) have been identified as key molecules with plant growth-stimulating activity that are capable of modulating physiological and metabolic processes in seeds, seedling, and plants (Hernández-Herrera et al., 2016; Mzibra et al., 2018; Hamouda et al., 2022; Ribeiro et al., 2024).

Both *S. liebmannii* and *U. ohnoi* often proliferate due to eutrophication, resulting in “green tides” caused by *Ulva* and “golden tides” caused by *Sargassum*, which can wash ashore in large volumes. The phenomena of green and golden tides pose environmental challenges while also offering valuable opportunities for resource utilization (Kidgell et al., 2019; Shukla et al., 2025). In intensive cultivation systems, the high biomass productivity of *U. ohnoi* is especially noteworthy, with Mata et al. (2016) reporting daily growth rates of up to 38 g dry weight (dw)·m⁻².

Among the conventional methods for preparing algal extracts, those involving pH variation are particularly important given their impact on the biochemical composition of the final product (Godlewska et al., 2017). Alkaline extraction is the most widely used approach, as it allows for high recovery of soluble polysaccharides and oligosaccharides while minimizing structural degradation (Briceño-Domínguez et al., 2014; Sharma et al., 2012). During this type of extraction, functional groups, such as hydroxyl, carboxyl, and sulfate, are converted into anionic forms, conferring a polyelectrolyte property to the extracted macromolecules (Reyes-Ortega, 2014). This property enables electrostatic interactions with positively charged LDH layers, creating opportunities to develop novel LDH-seaweed composites (Leroux et al., 2004; Nunes et al., 2020).

Several studies have demonstrated the successful incorporation of seaweed-derived polysaccharides into LDH matrices, producing composite materials with synergistic properties from both components with promising agricultural applications (Leroux et al., 2004; Nunes et al., 2020; de Castro et al., 2020). These nanocomposites integrate the structural and functional stability of LDHs with the biological activity of algal extracts, which offers a means to enhance natural plant processes and overall crop performance while creating opportunities to develop sustainable production strategies.

Although extensive research has been conducted on LDHs and seaweed extracts separately, there remains a research gap in understanding their potential synergistic effects when integrated into nanostructured composites for agricultural use. LDHs are known for their high stability and ability to provide controlled release of guest molecules, whereas seaweed extracts are rich in diverse of bioactive compounds with biostimulant properties. However, their integration as a strategy to enhance seed germination and seedling growth has yet to be systematically explored.

In this study, we synthesized and characterized composites based on Mg-Al LDHs and alkaline extracts of *S. liebmannii* and *U. ohnoi*, and evaluated their biological activity through seed germination and seedling growth assays in tomato (*Solanum lycopersicum*) and root induction assays in mung bean (*Vigna radiata*) cuttings. We hypothesized that LDH-seaweed composites can serve as next-generation biostimulants by promoting root and shoot development during the early stages of plant growth. To our knowledge, this is the first study to investigate LDH-seaweed composites as biostimulants for crop production, specifically by evaluating their effects on seed germination and early plant growth. The integration of nanotechnologies with natural marine resources, such as seaweeds, presents a promising approach for developing innovative phycobiostimulants capable of addressing current challenges in the transition toward sustainable agriculture.

## 2 Materials and methods

### 2.1 Algae material and seaweed extract preparation

Dry powder biomass of *U. ohnoi* and *S. liebmannii* was provided by the Biotechnology Research Laboratory of the University of Guadalajara (CUCBA). Seaweed extracts were prepared under alkaline conditions as described by Di Filippo-Herrera et al. (2021), with some modifications. Briefly, the dry powder of each seaweed (20 g) was rehydrated with constant stirring in distilled water (500 mL) for 15 min, and the pH was adjusted to 12 with potassium hydroxide (KOH). Subsequently, the two solutions were autoclaved at 121 °C for 15 min at 1.5 kg·cm^−2^. Subsequently, the hot alkaline extracts were filtered through Whatman No. 40 filter paper and stored at 4 °C until use. The biochemical characterization of the alkaline extracts of *Sargassum liebmannii* and *Ulva ohnoi* are showed in **Supplementary Table 1**.

### 2.2 Synthesis of LDH-seaweed composites

Magnesium-aluminum (Mg-Al) LDHs were prepared using the coprecipitation method. All chemicals [magnesium chloride hexahydrate (MgCl_2_·6H_2_O), aluminum chloride hexahydrate (AlCl_3_·6H_2_O), and sodium hydroxide (NaOH)] were purchased from Golden Bell reagents (Mexico) and had a reagent purity of 98%. In brief, a 40 mL solution was prepared using double-distilled deionized water and magnesium (1.43 g) and aluminum (0.85 g) salts at a Mg: Al molar ratio of 2. The pH was adjusted to 8.5 by the dropwise addition of 1 M NaOH under constant stirring at room temperature (~27 °C) for 30 min.

A molar ratio of 2 was chosen because it has been found to provide a higher positive charge density for the LDH nanosheets compared to higher ratios (Newman and Jones, 1998), which could provide a larger specific surface area (Matsuda et al., 2023) for interaction with the anions of the seaweed extracts. Subsequently, LDH-seaweed composites were prepared by mixing the 40 mL of the LDH suspension with 150 mL of the alkaline extract (either *Sargassum* or *Ulva*) under constant stirring for 60 min to promote the immobilization of the anionic compounds. The composites were recovered by centrifugation at 1,328 x *g* at 22 °C for 5 min using a Gyrozen 1248R centrifuge (Gyrozen Co., Ltd., South Korea), washed with deionized water up to a neutral pH, and lyophilized at –80 °C for 48 h. The solid material was ground in an agate mortar and stored in polypropylene tubes under vacuum in the dark. The materials were categorized as LDH, LDH-Sargassum, and LDH-Ulva.

### 2.3 LDH-seaweed composite characterization

The LDH-seaweed composites were characterized by different techniques, including X-ray diffraction (XRD), thermogravimetric analysis (TGA), differential scanning calorimetry (DSC), Fourier transform infrared spectroscopy (FT-IR), dynamic light scattering (DLS), scanning electron microscopy (SEM), and energy dispersive X-ray spectroscopy (EDS).

#### 2.3.1 X-ray diffraction

The XRD patterns were acquired using a PANalytical EMPYREAN diffractometer with Cu-Kα radiation at 40 kV and 30 mA. Scans were performed in the 2-theta mode (from 5 to 70°), with a step size of 0.02° and a scan rate of 30 s per step. The diffractograms of LDH-seaweed composites were compared with those of hydrotalcite and brucite obtained from an online database (https://www.crystallography.net/cod/).

#### 2.3.2 Thermal analysis

The thermal decomposition profile and physicochemical transitions of the LDH-seaweed composites were assessed by TGA and DSC, respectively, with 2 mg of powder heated at a rate of 20 °C per min under a nitrogen flow of 20 mL·min^−1^ in a Discovery thermogravimetric analyzer (TGA) and differential scanning calorimeter (DCS) (TA Instruments, New Castle, USA).

#### 2.3.3 Fourier transform infrared spectroscopy

The FT-IR analysis was performed in the attenuated total reflectance mode using an iS50 ATR spectrometer (Thermo Fisher Scientific, Waltham, USA) to identify the functional groups present in the mid-infrared region (4000–400 cm^−1^). Spectra were averaged from 15 scans with a resolution of 4 cm^−1^.

#### 2.3.4 Dynamic light scattering (DLS)

The size of the particles in the aqueous suspensions was determined via DLS in a Zetasizer Lab (Malvern Panalytical, Malvern, UK). Prior to measurement, 10 mg of the powder sample were dispersed in 3 mL of double-distilled deionized water and sonicated at 40 kHz with a maximum power of 100 W for 10 min, while maintaining the temperature at 25–28 °C.

#### 2.3.5 Scanning electron microscopy-energy dispersive X-ray spectroscopy

Composite morphology was explored using a QUANTA 250 environmental scanning electron microscope (ESEM) (FEI Company, Hillsboro, USA) in low vacuum mode. The samples were resuspended in water and sonicated under the same conditions as the DLS analysis. A drop of the suspension was then deposited onto a carbon tape-coated aluminum holder and allowed to dry at 25 °C. Micrographs were analyzed with a 15 kV electron beam. Moreover, EDS coupled to SEM equipment was used to determine the elemental composition of the sample surface.

### 2.4 Tomato germination and seedling growth assays

Seed germination was performed with certified tomato seeds (*Solanum lycopersicum* cv. Rio Grande; Kristen seed^®^, San Diego, USA) according to the Association of Official Seed Analysts (Association of Official Seed Analysts, 2005). Tomato seeds were initially surface-disinfected in 3% sodium hypochlorite (NaClO) solution for 5 min and subsequently triple-rinsed in sterile double-distilled water for 1 min each. Aqueous suspensions of LDH or composites (LDH-Sargassum and LDH-Ulva) were prepared at different concentrations (0 mg·mL^−1^, 1.57 mg·mL^−1^, 3.14 mg·mL^−1^, 6.28 mg·mL^−1^, 12.56 mg·mL^−1^, 25.12 mg·mL^−1^, or 50.25 mg·mL^−1^) by dispersing the powder samples in double-distilled deionized water. The suspensions were then sonicated in an ultrasonic bath operating at 40 kHz and 100 W for 10 min at room temperature. Afterward, the seeds were immersed in 3 mL of LDH or composite suspensions for 24 h under constant stirring at room temperature to ensure homogeneous exposure to the treatments. The 0 mg·mL^−1^ concentration (distilled water) was considered a control group. The seeds from each treatment were arranged between two sheets of germination paper (25 × 38 cm) and then wrapped to form rolls. The rolls were placed vertically inside a plastic bag and maintained at 26 ± 2 °C with approximately 95% relative humidity for 8 days. A total of 100 seeds for each treatment were divided into three replications. Germination was considered only when the length of the radicle was at least 2 mm.

The germination percentage (GP) was determined using Equation 1:


(1) $$\text{GP}\;\left(\%\right)=\frac{\text{Number of germinated seeds}}{\text{Total number of seeds}}\;\times 100$$

As described by Abdul Baki and Anderson (1973) and Zhang et al. (2020), respectively, seed vigor was calculated using vigor index-1 (VI-1) (Equation 2) and vigor index-2 (VI-2) (Equation 3):


(2) $$\begin{array}{c} \text{Vigor index }1\;\left(VI-1\right)\\ =\;\text{Germination}\;(\%)\;\times \;\text{Seedling length}\;(cm) \end{array}$$

And


(3) $$\begin{array}{c} \text{Vigor index}\;2\;(VI-2)\\ =\;\text{Germination}\;(\%)\;\times \;\frac{\text{Fresh weight of seedlings}\;(g)}{\text{Number of seedlings}} \end{array}$$

To evaluate post-germinative growth, twelve 13-day-old seedlings from each replicate were randomly selected. Morphological measurements, including hypocotyl length, radicle length, and total seedling length, were obtained using ImageJ v1.52a software (https://imagej.nih.gov/ij/download.html). In addition, the fresh and dry weights of the seedlings were recorded with an analytical balance A&D HR-200 (Data Weighing Systems, Inc. Wood Dale, IL. USA). All biological descriptors were averaged per replicate (*n* = 3) for statistical analyses.

### 2.5 Mung bean rooting assays

The mung bean *Vigna radiata* (L.) Wilczek model described by Lötze and Hoffman (2016) was utilized to evaluate the rooting activity of LDH and the composite suspensions (LDH-Sargassum and LDH-Ulva). Mung bean seeds were sown in a germination tray containing a mixture of peat moss (Sunshine Mix 3™) and vermiculite in a ratio of 1:1 (v/v). The seedlings were maintained in a growth chamber at 26 ± 2 °C and relative humidity of ~85%, with a 16:8 light: dark photoperiod (100–155 μm·s^−1^·m^−2^) and watered as required. After 9 days, seedlings that had reached 12 cm in height and developed their first two true leaves were cut to 3 cm below the cotyledons, and the cotyledons were removed.

The composite suspensions (LDH-Sargassum and LDH-Ulva) were prepared as described in Section 2.4, at concentrations of 1.57 mg·mL^−1^, 3.14 mg·mL^−1^, and 6.28 mg·mL^−1^, selected based on the best results obtained in tomato germination assays in this study. The stem cuttings were transferred to 15-mL centrifuge tubes covered with Parafilm^®^ (holes were pierced in the film), with 4 cm of the cuttings immersed in 5 mL of previously sonicated test suspensions. Two cuttings were placed in each tube, and six replicate tubes were included per treatment. Cuttings were maintained for 12 days under the growth conditions described above. At the end of the experiment and after drying in an oven at 60 °C overnight, the number and dry weights of adventitious roots were recorded. In addition, one cutting per tube was randomly selected, and the lengths of five roots per cutting were measured. The values for each biological descriptor were averaged per tube to obtain the mean of the true replicate (*n* = 6).

### 2.6 Statistical analysis

The experimental units were completely randomized to create a two-way design with crossed and fixed factors (model type I). The first factor was treatment (LDH, LDH-Sargassum, or LDH-Ulva), and the second factor was concentration (0 mg·mL^−1^, 1.57 mg·mL^−1^, 3.14 mg·mL^−1^, 6.28 mg·mL^−1^, 12.56 mg·mL^−1^, 25.12 mg·mL^−1^, and 50.25 mg·mL^−1^). This experimental design was analyzed using a permutational multivariate analysis of variance (PERMANOVA) constructed with a Euclidean distance matrix; the data were normalized to Z values (or zero-mean standardization). The significance of the PERMANOVA model (α = 0.05) was tested with 10,000 permutations of the residuals under a reduced model and sum of squares type III. The experimental design terms that were statistically significant in the PERMANOVA global test were further examined with *post hoc* pairwise comparisons, using the pseudo-t statistic and *p*-values estimated by the Monte Carlo procedure due to the limited number of possible permutations (≤ 100). A test for homogeneity of multivariate dispersions (PERMDISP) was conducted to assess the dispersion among treatments and concentrations (Anderson, 2006). A principal coordinate analysis (PCO) ordination was also used to visualize patterns, in which biological descriptors were represented as vectors based on a multiple correlation analysis and a correlation circle. The PERMDISP and the PCO ordination were constructed using the same data pretreatment and resemblance coefficient as the PERMANOVA model. All statistical analyses were performed in PRIMER + PERMANOVA v.7.0.13 software, while data from XRD, TGA, DCS, FT-IR, DLS, and EDS analyses were plotted and analyzed using OriginPro 2018 v. 95E (https://www.originlab.com/2018).

## 3 Results

### 3.1 Characterization of composites

#### 3.1.1 X-ray diffraction

**Figure 1:**
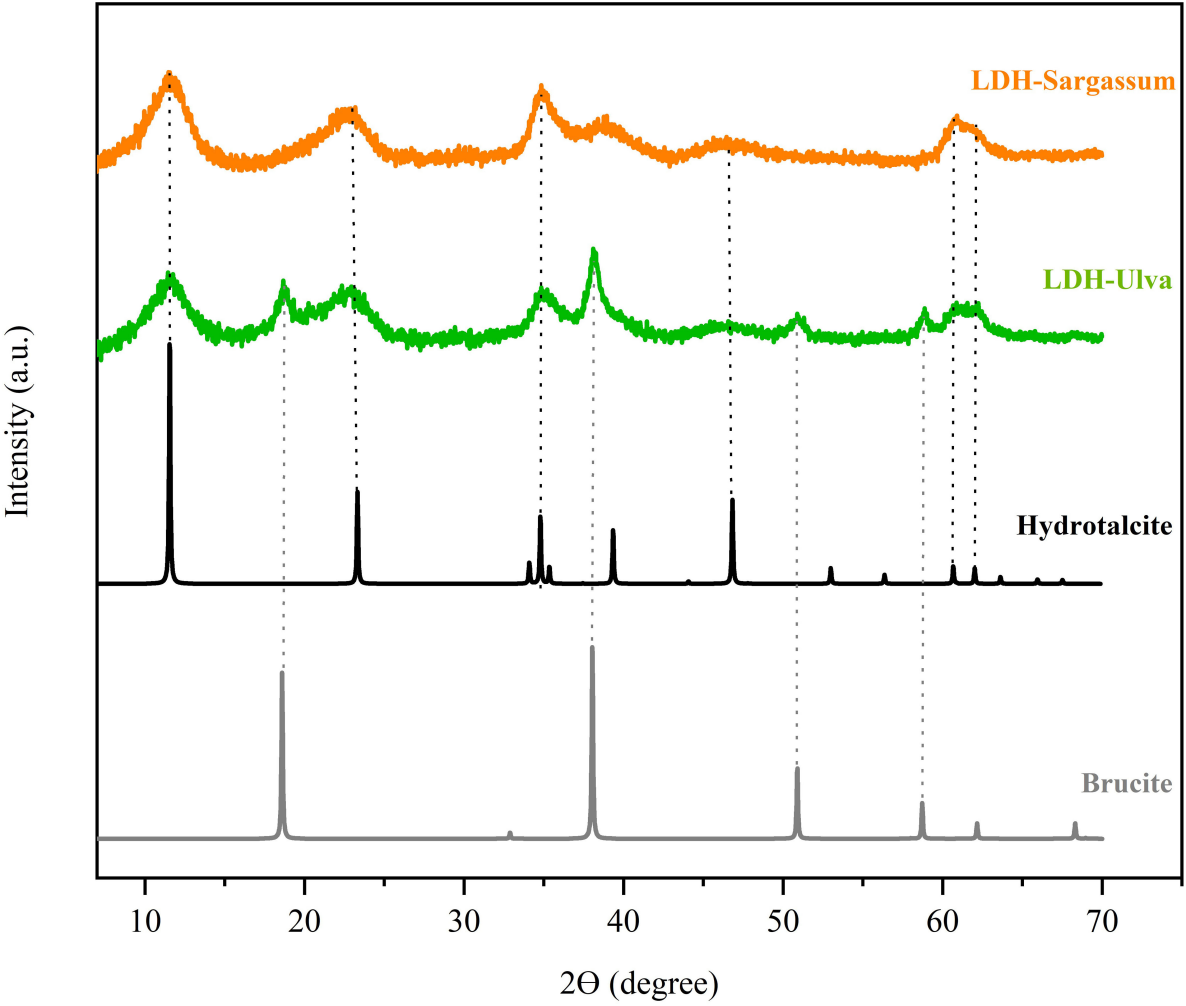
X-ray diffraction (XRD) patterns of the layered double hydroxide (LDH)-Sargassum and LDH-Ulva composites and the reference samples (hydrotalcite, [ID 81963] and brucite [ID 1000054]) obtained from an online database (https://www.crystallography.net/cod/).

All XRD patterns showed characteristic hydrotalcite peaks at 2-theta values at 11.5°, 23.2°, 34.8°, 46.6°, 60.7°, and 62.0° (**Figure 1**), which can be attributed to the diffraction planes (0 0 3), (0 0 6), (0 1 2), (0 1 8), (1 1 0), and (1 1 3), respectively. Furthermore, the LDH-Ulva diffraction pattern showed weak peaks of brucite [Mg(OH)_2_] at 18.5°, 38.18°, 50.9°, and 58.7°, indicating the formation of a second phase (**Figure 1**).

#### 3.1.2 Thermal analysis

**Figure 2:**
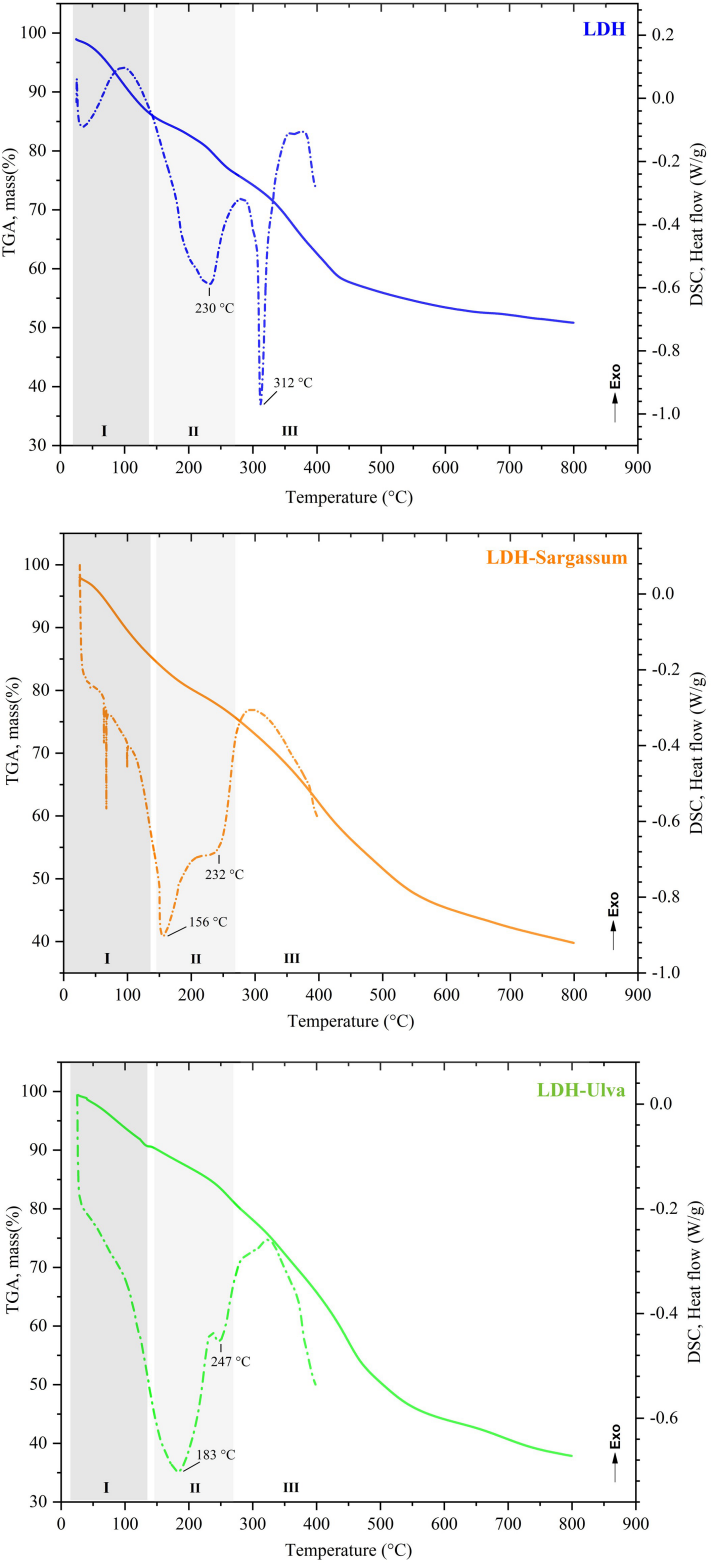
Thermal analysis of the Mg-Al layered double hydroxides (LDH) and the LDH-Sargassum and LDH-Ulva composites. The dashed lines correspond to thermogravimetric analysis (TGA). The dotted lines correspond to differential scanning calorimetry (DSC) analysis.

The TGA profiles and DSC curves are shown in **Figure 2**. The samples exhibited differences in the shape and intensity of the thermal events grouped in regions I, II, and III.

The decomposition profile in the TGA curve of LDH indicated a total mass loss of 49.14% (**Figure 2**). Region I comprised events occurring at room temperature to around 140 °C (initial mass loss of 13.75%), with no clear DSC events. Region II comprised events occurring at 140 °C to 275 °C (mass loss of 10.41%) and contained a DSC event at 230 °C. Finally, region III corresponded to the last decomposition event, including a DSC event at 312 °C.

The thermal decomposition behavior of LDH-Sargassum and LDH-Ulva displayed changes in the DSC profiles in region II, likely due to the chemical constituents of the seaweeds. In contrast, in the TGA plots, the LDH-Sargassum and LDH-Ulva composites showed a total mass loss of 60.15% and 62.12%, respectively, at the end of the thermal decomposition, representing an average increase of 12% in mass loss compared to the pristine LDH sample, which was attributed to the content of seaweed extract in the composites. The first mass loss occurred at temperatures ranging from 25 to 140 °C. This step was followed by another step ending at 275 °C, associated with two thermal DSC events occurring at different temperatures and intensities (156 and 232 °C for LDH-Sargassum and 183 and 247 °C for LDH-Ulva). The most significant mass loss was observed above 300 °C, reaching 35.33% for LDH-Sargassum and 42.76% for LDH-Ulva. The results confirm that the LDH-seaweed composites are composed of an inorganic phase corresponding to the Mg-Al LDHs and an organic fraction derived from the seaweed extracts.

#### 3.1.3 Fourier-transform infrared spectroscopy

**Figure 3:**
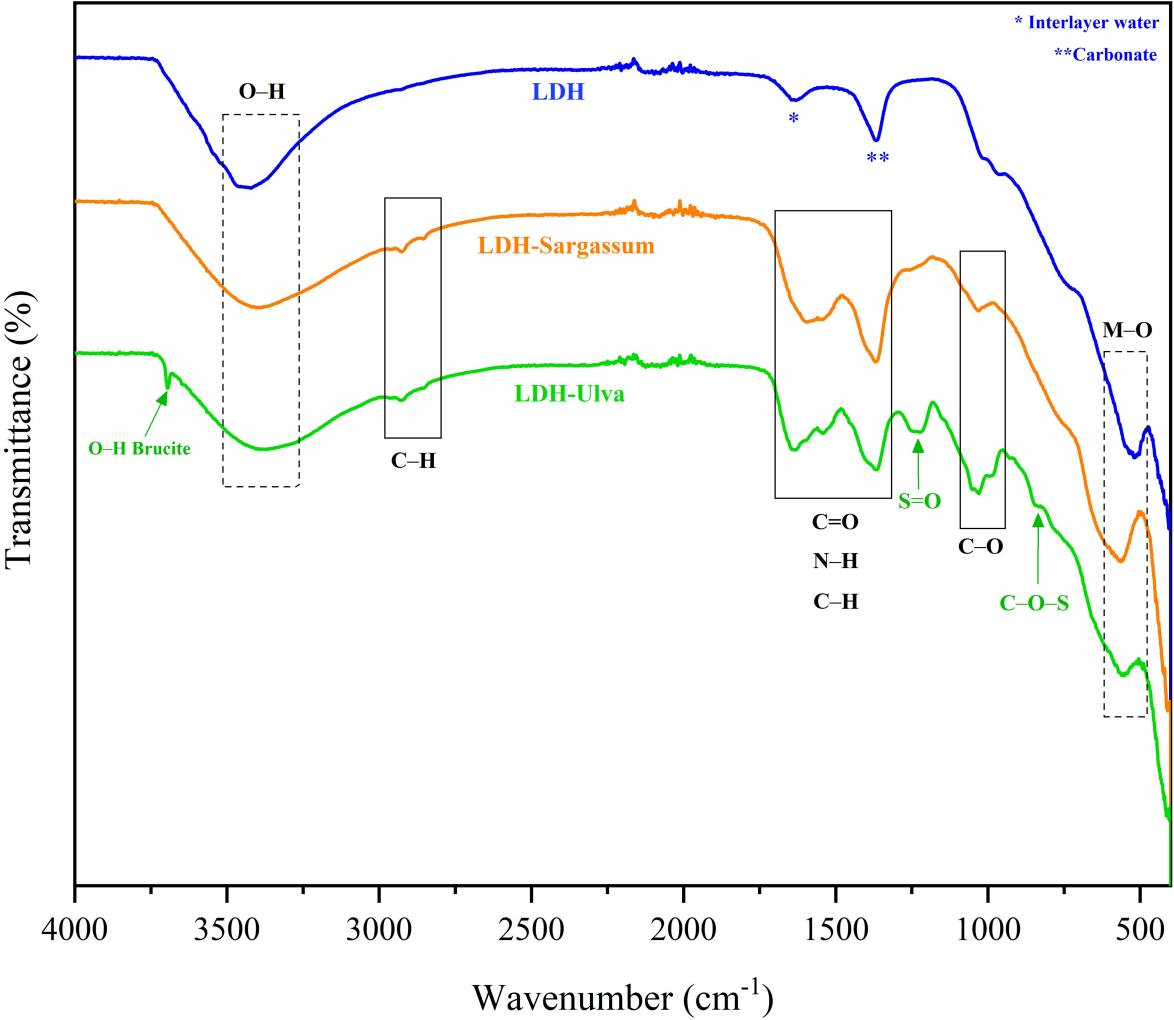
Infrared spectra of the Mg-Al layered double hydroxide (LDH) and the LDH-Sargassum and LDH-Ulva composites. Signals within the boxes delineate by dotted lines correspond to Mg-Al LDH, while those in the boxes delineated with solid lines correspond to the seaweed compounds.

In the FT-IR spectrum of pristine LDH (**Figure 3**), the broad and intensive bands between 3500 and 3250 cm^−1^ corresponded to the stretching vibration of the hydroxyl group (O–H) of the hydroxylated layers and water molecules in the interlayer region. The band at 1634 cm^−1^ was associated with the angular deformation vibration of water (H_2_O), while the strongest absorption signal at 1368 cm^−1^ was attributed to carbonate stretching, which is formed from the atmospheric CO_2_ during synthesis. The adsorption bands in all samples below 800 cm^−1^ were associated with metal-oxygen (M–O) and metal-oxygen-metal (O–M–O) vibrations.

The infrared spectra of the LDH-seaweed composites showed a combination of bands from the pristine LDH and seaweed extract constituents (**Figure 3**). The spectrum of LDH-Sargassum contained bands for O–H stretching (at 3394 cm^−1^), aliphatic C–H stretching (at 2924 and 2849 cm^−1^), C=O stretching (at 1600 cm^−1^), and C–O–C stretching vibrations (1030 cm^−1^). In the spectrum of LDH-Ulva, bands corresponding to O–H stretching (at 3365 cm^−1^), aliphatic C–H stretching (at 2924 and 2849 cm^−1^), C=O stretching (at 1637 cm^−1^), N–H stretching (at 1535 cm^−1^), S=O stretching vibrations in the sulfate group (at 1232 cm^−1^), C–O–C stretching vibrations (at 1030 cm^−1^), and C–O–S bending of the sulfate in axial position (at 846 cm^−1^) were observed. Moreover, a characteristic narrow band of brucite at 3698 cm⁻¹ was detected in the LDH-Ulva sample, supporting the XRD signals for this material. Lastly, a band at 1365 cm⁻¹ was present in both composites (**Figure 3**), which was associated with carbonate ions similar to those in pristine LDH. This signal possibly overlapped the C=O stretching band that typically appeared in the region of 1400–1425 cm⁻¹.

#### 3.1.4 Dynamic light scattering

**Figure 4:**
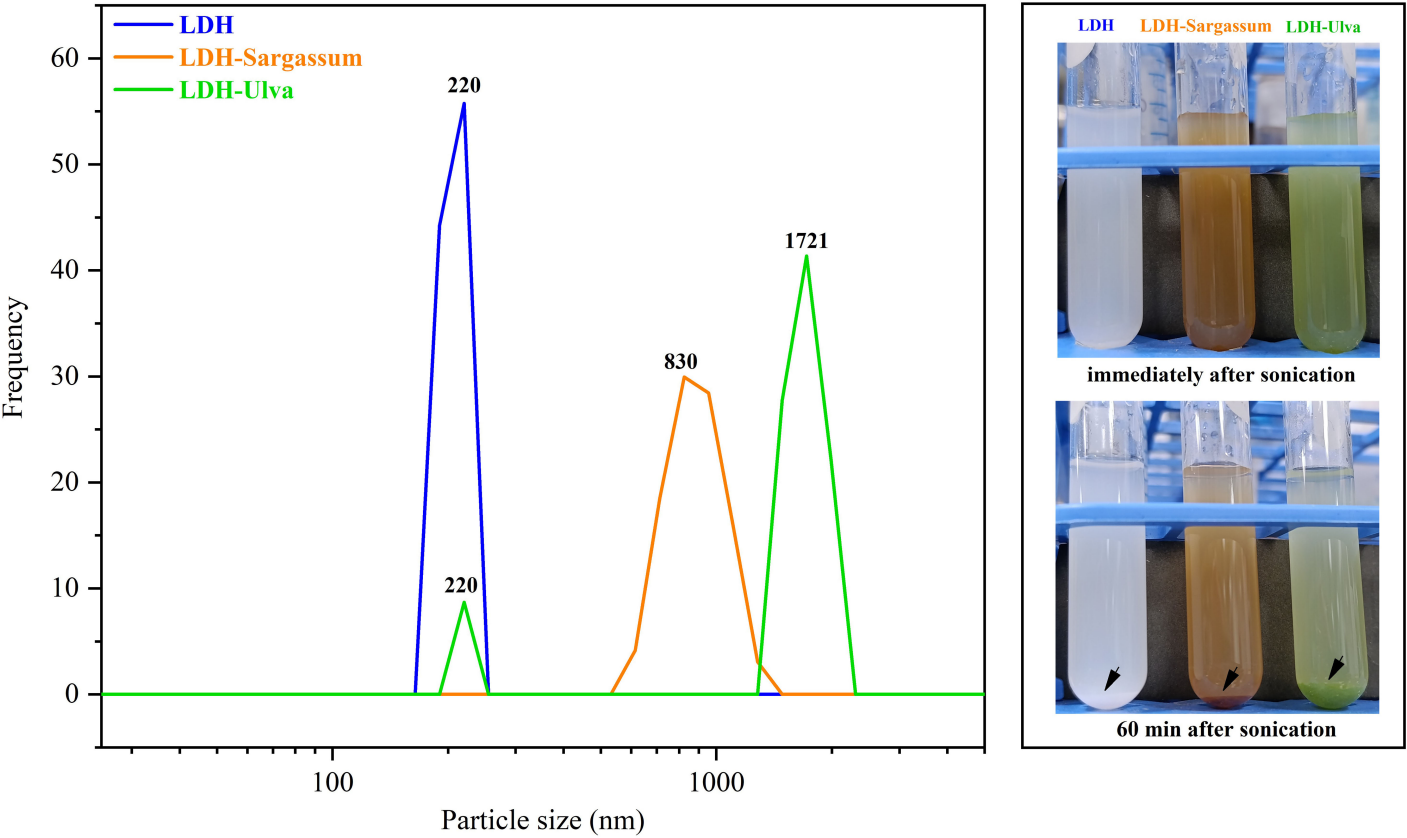
Particle size distribution in aqueous suspensions of the Mg-Al layered double hydroxides (LDH) and the LDH-Sargassum and LDH-Ulva composites. The images on the right depict the samples in aqueous suspensions immediately following 60 min of sonication, during which precipitation occurred.

**Figure 4** depicts the particle size in the aqueous suspension of the composites and pristine LDH. The hydrodynamic particle size of LDH and LDH-Sargassum was 220 and 825 nm, respectively. In the case of LDH-Ulva, two distinct particle size populations were observed, a less abundant population of 221 nm and a predominant population of 1721 nm, which remained large even after ultrasonication for 10 min. This observation could be due to the coexistence of brucite and hydrotalcite-like particles. In addition, the aqueous suspensions of all samples exhibited visible particle aggregation and precipitation over time, as shown in **Figure 4**, which may have contributed to increased hydrodynamic size.

#### 3.1.5 Scanning electron microscopy-energy dispersive X-ray spectroscopy

**Figure 5:**
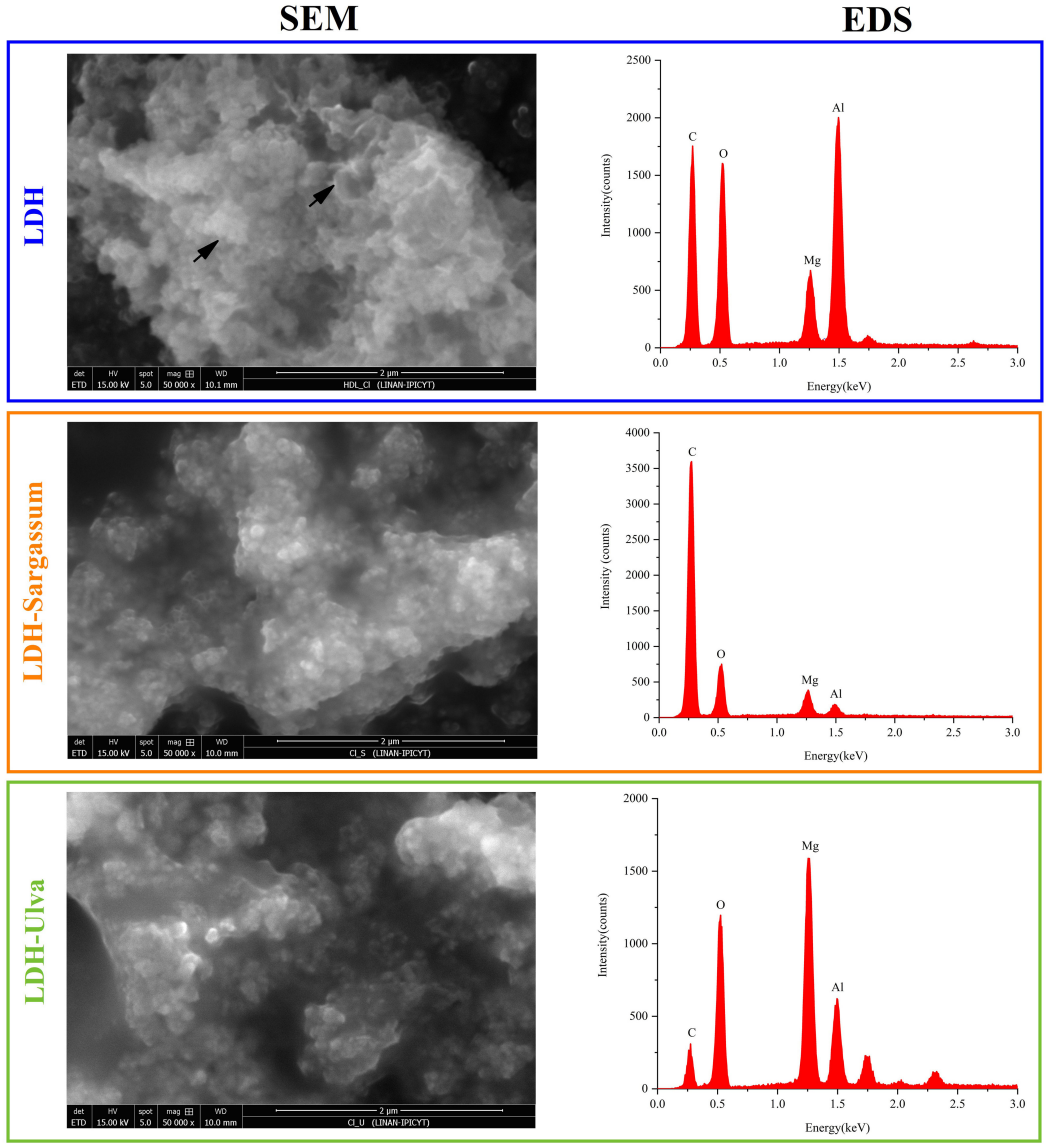
Scanning electron microscopy (SEM) and energy dispersive X-ray spectroscopy (EDS) of the Mg-Al layered double hydroxides (LDH) and the LDH-Sargassum and LDH-Ulva composites. The samples were resuspended in water, sonicated, and deposited onto a carbon tape for analysis. Micrographs were obtained at a magnification of 50,000×.

SEM micrographs were utilized to explore the morphology of pristine LDH and the LDH-seaweed composite samples (**Figure 5**). The LDH image revealed morphology characteristic of LDH particles, with aggregated thin flakes with well-defined edges that were randomly arranged and formed structures similar to the “desert rose” type arrangement (**Supplementary Figure 1**). This flake morphology indicated effective organization of the layered structure. Aggregates of particles with a granular appearance were also discernible. The flake and granular aggregates coalesced to form larger secondary particles, generating cavities. In contrast, a higher degree of aggregation was observed in the micrographs of the LDH-Sargassum and LDH-Ulva composites than in the pristine LDH sample, with aggregation being more pronounced in LDH-Sargassum.

In addition, an EDS analysis was conducted to evaluate elemental composition (**Figure 5**). In all cases, the elements detected were oxygen (O), magnesium (Mg), and aluminum (Al), which were fundamental constituents of the LDH structure. The carbon detected in the samples was not considered in the EDS analysis, as the carbon tape interferes with quantification.

### 3.2 Effects on tomato seed germination and seedling growth

**Figure 6:**
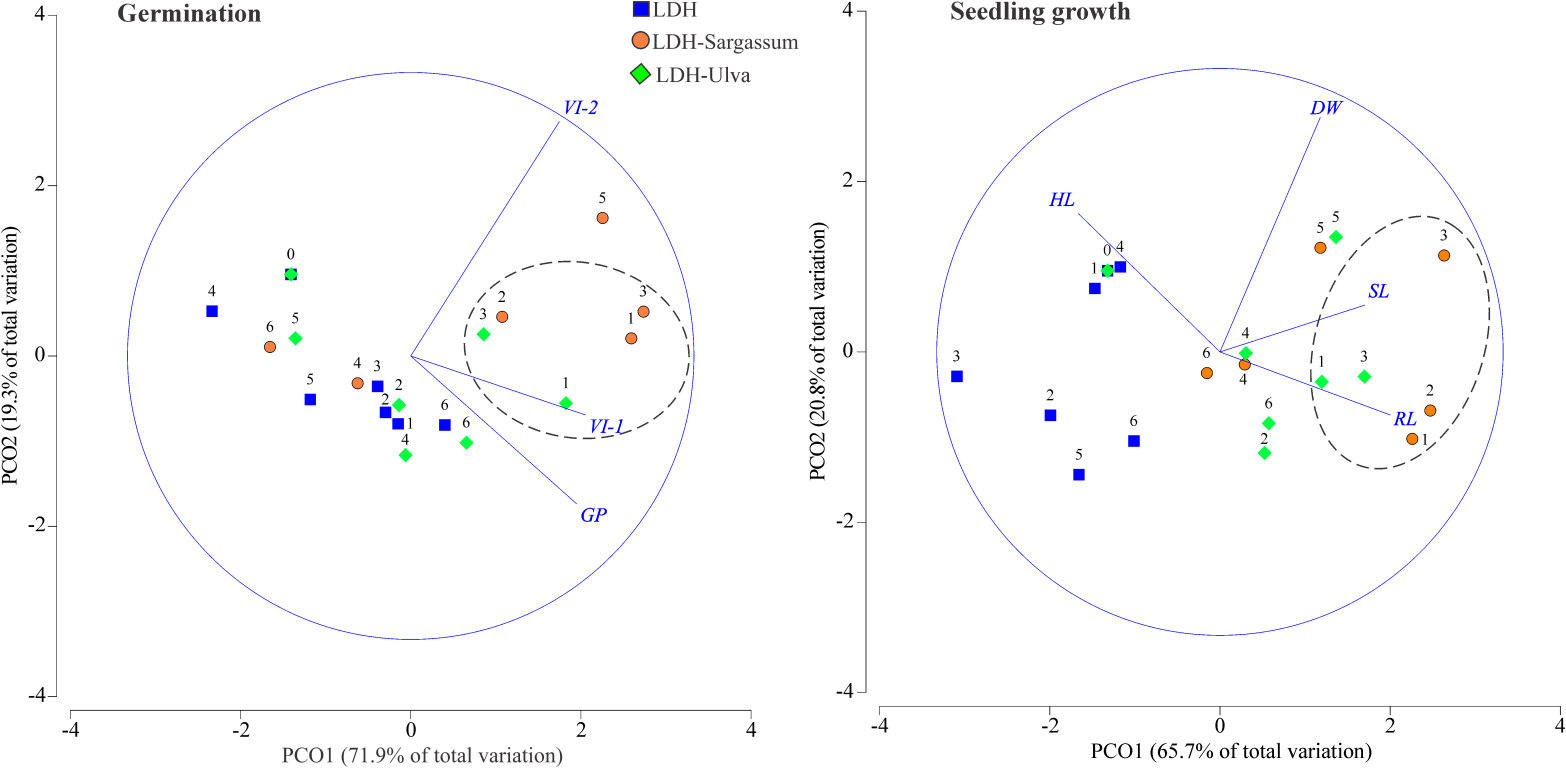
Principal coordinate (PCO) analysis ordinations of variables related to tomato seed germination and seedling growth in response to different treatments (LDH, LDH-Sargassum, and LDH-Ulva) and concentrations [0, 1.57 (1), 3.14 (2), 6.28 (3), 12.56 (4), 25.12 (5), and 50.25 (6) mg·mL^−1^]. Percentage germination (GP), vigor index-1 (VI-1), vigor index-2 (VI-2), hypocotyl length (HL), radicle length (RL), seedling length (SL), and dry weight (DW).

Multivariate approaches were used to analyze the biological activity of LDH and LDH-seaweed composites during tomato germination and seedling emergence. **Supplementary Table 2** shows the average values of all variables measured in the experiments, while the effects of treatment with LDH and the LDH-seaweed composites (LDH-Sargassum or LDH-Ulva) at various concentrations are presented in **Table 1** and **Figure 6**.

**Table 1:** PERMANOVA and PERMDISP outputs for tomato germination and seedling growth.

Source	PERMANOVA			PERMDISP	
	Pseudo-F^1^	*P*(perm)^2^	C.V.(%)^3^	F	*P*(perm)^2^
*Germination*					
Concentration (A)	4.92	<0.001	23.3	2.58	0.056
Treatment (B)	7.25	<0.001	19.3	4.31	0.023
AxB	2.18	<0.001	22.2	2.01	0.544
Residual			35.2		
*Seedling growth*					
Concentration (A)	6.46	<0.001	18.8	7.04	<0.001
Treatment (B)	32.50	<0.001	29.6	1.26	0.306
AxB	4.86	<0.001	27.4	3.02	0.153
Residual			24.2		

Results showed significant differences (*P* ≤ 0.05) based on 10,000 permutations. ^1^Pseudo-F = multivariate analog of Fisher’s F statistic (i.e., ratio of variance). ^2^P(perm) = Permutational *P* value (i.e., the proportion of permuted pseudo-F statistics ≥ the original pseudo-F). ^3^C.V. (%) = component of variation in percentage.

The two-way crossed PERMANOVA outputs of both germination and seedling growth revealed significant differences among treatments (LDH, LDH-Sargassum, and LDH-Ulva), concentrations (0, 1.57, 3.14, 6.28, 12.56, 25.12, and 50.25 mg·mL^−1^), and their interaction, with 64.8% and 75.8% of variance explained in both models, respectively (*P* ≤ 0.001; **Table 1**). Consequently, the interaction was used as the main term for pairwise comparisons and interpretation of the results based on Monte Carlo *P*-values (*P*(MC), **Supplementary Table 3** and **Supplementary Table 4**). Furthermore, PERMDISP revealed no significant differences in the interaction for germination and seedling growth (*P* = 0.544 and *P* = 0.153, respectively; **Table 1**), demonstrating that variability within the dataset had a location effect, which suggests that dispersion was not the primary cause of the observed differences in these early stages of growth.

The pairwise comparisons of germination data indicated that the lowest concentrations (1.57 and 6.28 mg·mL^−1^) of both LDH-Ulva and LDH-Sargassum had a significant effect (0.008< *P*(MC)< 0.044) compared to the control (0 mg·mL^−1^). A similar result was observed when seeds were treated with 50.25 mg·mL^−1^ of LDH-Ulva (*P*(MC) = 0.019). Furthermore, LDH-Sargassum at 1.57 and 6.28 mg·mL^−1^ and LDH-Ulva at 1.57 and 12.56 mg·mL^−1^ promoted significant effects (0.019< *P*(MC)< 0.050) on germination compared to those of pristine LDH. Statistical differences (0.035< *P*(MC)< 0.044) were also found between LDH-Sargassum and LDH-Ulva at concentrations of 6.28 and 50.25 mg·mL^−1^ (**Supplementary Table 3**).

The pairwise comparisons for seedling growth indicated statistical differences (0.001< *P*(MC)< 0.027) between all concentrations of both LDH-seaweed composites (except for 50.25 mg·mL^−1^of LDH-Sargassum) and the control (0 mg·mL^−1^). Similarly, concentrations of 1.57, 3.14, 6.28, and 25.12 mg·mL^−1^ of both composites significantly impacted seedling growth (0.001< *P*(MC)< 0.042) compared to those of pristine LDH. In addition, a statistically significant difference (*P*(MC) = 0.03) was observed between LDH-Sargassum and LDH-Ulva at a concentration of 6.28 mg·mL^−1^ (**Supplementary Table 4**). Thus, LDH-seaweed composites promoted significant changes in germination and seedling growth compared to the control and pristine LDH.

The PCO analysis based on seed germination data showed that the first two axes explained 91.2% of the total variation (**Figure 6**). Axis 1 (PCO1) accounted for 71.9% of the observed variation and was mainly correlated with vigor index-1 (VI-1) and the germination percentage (GP). Axis 2 (PCO2) explained 19.3% of the variation and was mainly correlated with vigor index-2 (VI-2). The PCO ordination showed a clear separation between the three lowest concentrations of both LDH-seaweed composites (except for 3.14 mg·mL^−1^ of LDH-Ulva) and the remaining concentrations of the treatments. This pattern was consistent with the fact that the concentration factor explained the highest percentage of the observed variation in germination (23.3%; **Table 1**). Overall, lower concentrations of LDH-Sargassum and LDH-Ulva contributed to higher vigor index-1 values that increased by up to 21 and 17% at 6.28 mg·mL^−1^, respectively, compared to the control (**Figure 6**; **Supplementary Table 2**).

A second PCO analysis for seedling growth data explained 86.5% of the total variation, with 65.7% attributed to axis 1 (PCO1) and 20.8% attributed to axis 2 (PCO2) (**Figure 6**). PCO1 exhibited stronger correlations with radicle length and seedling length, while PCO2 was correlated with dry weight and hypocotyl length. The PCO ordination showed distinct grouping patterns based on the treatments, with a marked separation between the LDH-seaweed composite concentrations and those of pristine LDH. In this regard, the PERMANOVA model showed that the treatment factor accounted for 29.6% of the observed variation in seedling growth (**Table 1**). Another important observation was that seedling radicles and hypocotyls exhibited distinct responses to composites. Specifically, lower concentrations of LDH-Sargassum (1.57 and 3.14 mg·mL^−1^) and LDH-Ulva (1.57 and 6.28 mg·mL^−1^) were strongly associated with an increase in radicle length of up to 47–53% and 33–42%, respectively, compared to the control, although they were negatively correlated to hypocotyl length (**Figure 6**; **Supplementary Table 2**). Lastly, an 11% increase in SL was observed at 6.28 mg·mL^−1^ of the LDH-Sargassum (**Figure 6**; **Supplementary Table 2**).

**Figure 7:**
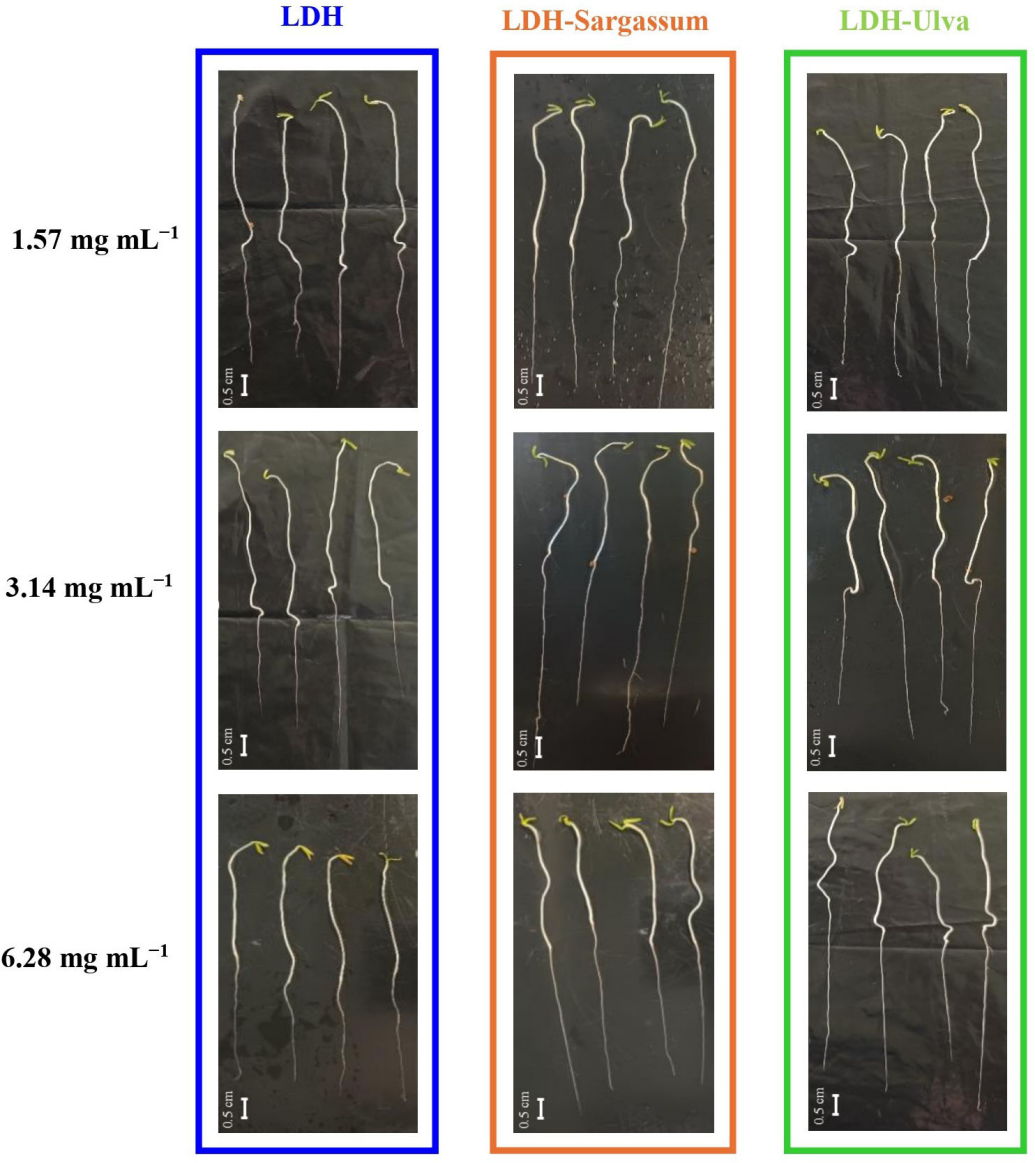
Tomato seedlings (13-day-old) treated with Mg-Al layered double hydroxide (LDH) and LDH-seaweed composites at concentrations of 1.57, 3.12, and 6.28 mg·mL^−1^. Bar = 0.5 cm.

The results indicate that the lowest concentrations of 1.57, 3.14, and 6.28 mg·mL^−1^of the LDH-seaweed composites enhanced seed vigor, early seedling growth, and radicle elongation in particular under laboratory conditions (**Figure 7**). Therefore, we decided to evaluate rooting promotion using a mung bean cutting bioassay.

### 3.3 Effects on mung bean rooting

**Figure 8:**
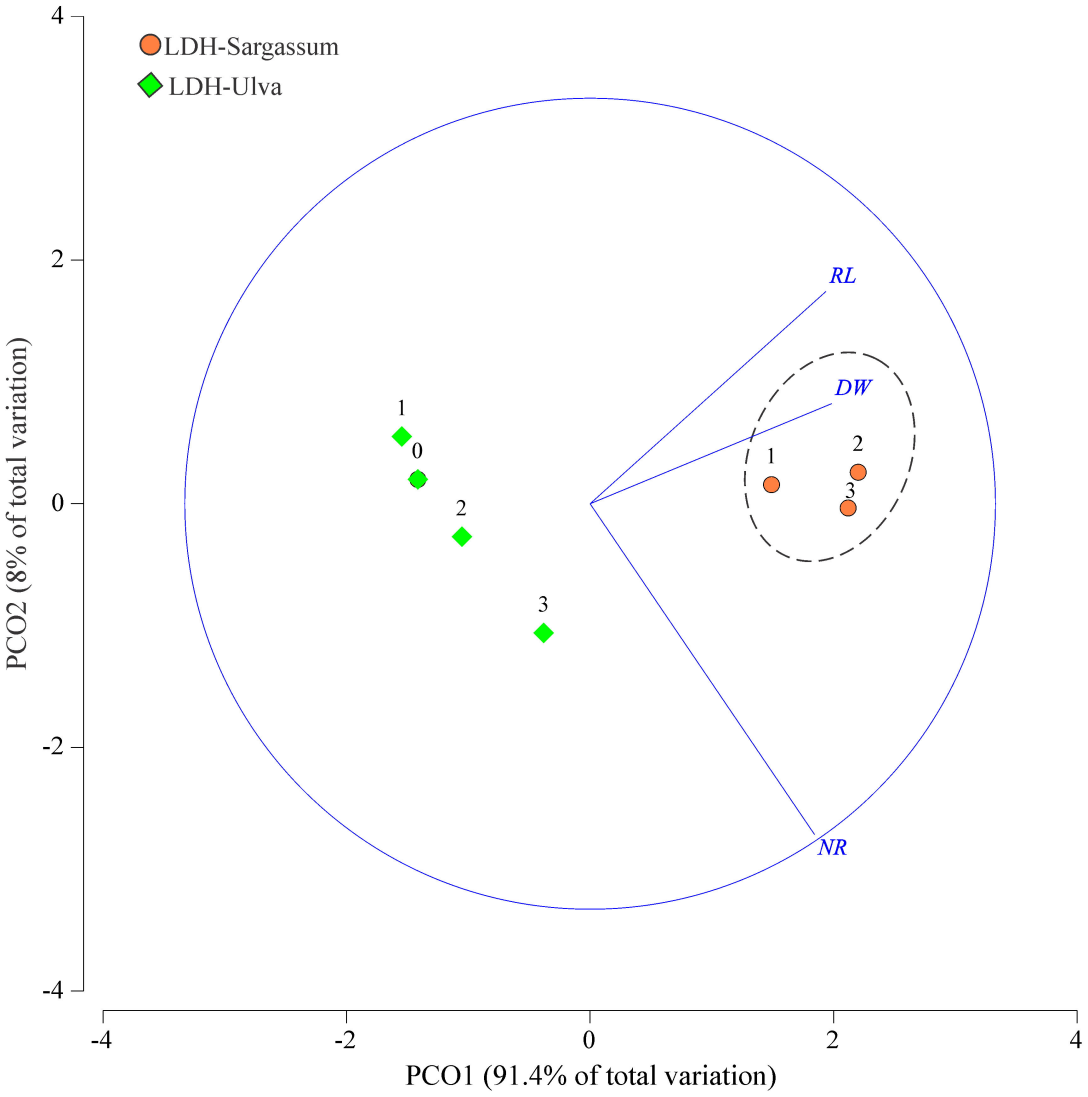
Principal coordinate (PCO) ordination of the variables related to root formation in mung bean cuttings in response to different treatments (LDH-Sargassum and LDH-Ulva) and concentrations [0, 1.57 (1), 3.14 (2), and 6.28 (3) mg·mL^−1^]. Root length (RL), dry weight (DW), and number of roots (NR).

In the mung bean bioassay, the ability of the LDH-seaweed composites to promote rooting at 1.57, 3.14, and 6.28 mg·mL^−1^ was analyzed using a multidimensional approach (**Table 2**; **Figure 8**). The PERMANOVA outputs indicated that both factors (concentration and treatment) and their interaction showed a significant effect on rooting in mung bean cuttings (*P* < 0.001; **Table 2**). The PERMDISP results showed no significant differences for the interaction factor, indicating a location effect and that data dispersion was not the primary driver of the observed differences in rooting activity (*P* = 0.182; **Table 2**). The results of the pairwise comparisons at the interaction level indicated statistically significant differences (0.001< *P*(MC)<0.038) in adventitious root formation between the evaluated concentrations of both LDH-seaweed composites and the control (0 mg·mL^−1^). Furthermore, statistical differences (*P*(MC)< 0.001) in rooting were found within and between the concentrations of each treatment group (**Supplementary Table 5**).

**Table 2:** PERMANOVA and PERMDISP outputs for mung bean rooting.

Source	PERMANOVA			PERMDISP	
	Pseudo-F^1^	*P*(perm)^2^	C.V.(%)^3^	F	*P*(perm)^2^
Concentration (A)	120.87	<0.001	26.17	141.10	<0.001
Treatment (B)	501.14	<0.001	37.80	11.33	0.012
AxB	68.35	<0.001	27.74	1.89	0.182
Residual			8.28		

Results showed significant differences (*P* ≤ 0.05) based on 10,000 permutations.^1^Pseudo-F = multivariate analog of Fisher’s F statistic (i.e., ratio of variance). ^2^P(perm) = Permutational *P* value (i.e., the proportion of permuted pseudo-F statistics ≥ the original pseudo-F). ^3^C.V. (%) = component of variation in percentage.

**Figure 9:**
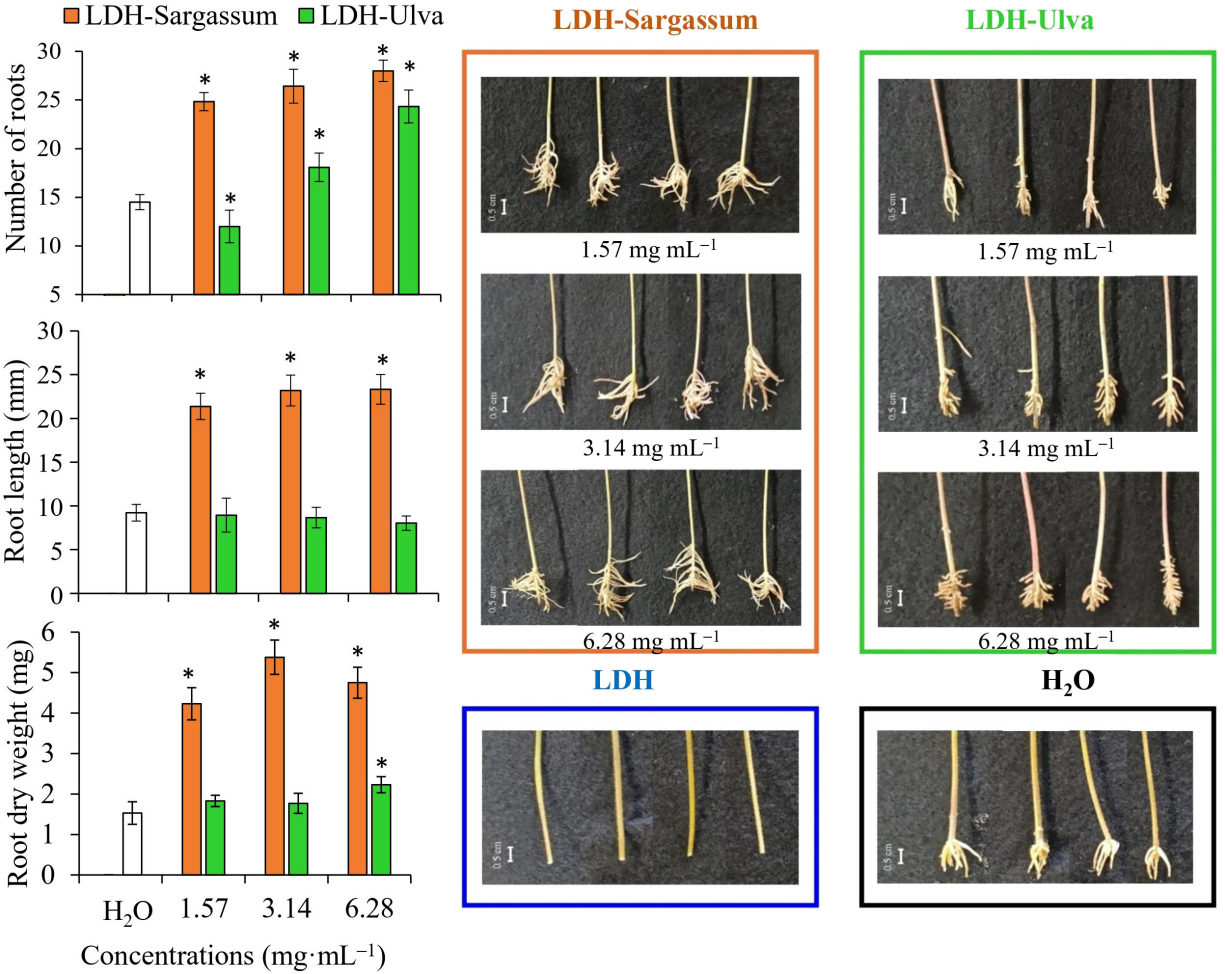
Adventitious root formation in mung bean cuttings following a 12-day treatment period with Mg-Al layered double hydroxide (LDH) and LDH-seaweed composites. Bar = 0.5 cm. Vertical bars show the means ± standard deviations (*n* = 6, each replicate consisted of two cuttings). Asterisks (*) indicate significant differences of each treatment with respect to the control (distilled water), based on pairwise PERMANOVA with Monte Carlo *p*-values (*P*(MC) ≤ 0.05).

By correlating the rooting variables with the LDH-Sargassum and LDH-Ulva concentrations through a PCO analysis, the first two axes accounted for 99.4% of the total variation (**Figure 8**). PCO1 explained 91.4% of the variation and was correlated with root dry weight and root length, while PCO2 explained only 8.0% and was mainly related to the number of roots formed in the mung bean cuttings. In the PCO ordination, LDH-Sargassum and LDH-Ulva were distinctly separated, supporting the statistical results that identified the treatment factor as the main contributor to the observed variation (37.8% of the total variation; **Table 2**). A closer clustering was observed among the LDH-Sargassum concentrations, which were strongly associated with root dry weight (**Figure 8**). Root dry weight in LDH-Sargassum was four-fold greater than the control (**Figure 9**).

The LDH-seaweed composites promoted root formation more extensively along the cuttings; however, LDH-Sargassum was more effective in enhancing the number of roots and root length, which ultimately led to greater biomass accumulation (dry weight). This effect was particularly evident with the concentration of 3.14 mg·mL^−1^. In contrast, LDH-Ulva increased the number and dry weight of roots at a concentration of 6.28 mg·mL^−1^. Lastly, the cuttings immersed in pristine LDH exhibited suppressed root formation, whereas roots in the distilled water control only appeared at the base of the cuttings (**Figure 9**).

## 4 Discussion

Recent studies have investigated the functionalization of LDHs with agriculturally relevant substances to improve plant growth and soil health (Singha Roy et al., 2022; Komarova et al., 2023). Building on this approach, we combined LDHs with extracts from *Sargassum liebmannii* and *Ulva ohnoi* to enhance the stability and delivery of seaweed-derived bioactives. Chemical analysis of the extracts (**Supplementary Table 1**) reveled high levels of organic matter, minerals, and secondary metabolites, such as phenolics and flavonoids, known for their antioxidant activity, stress mitigation, and growth-promoting effects. Compositional differences between the brown (*Sargassum*) and green (*Ulva*) algae suggest species-specific contributions to the observed biological effects. These findings provide a biochemical rationale for the use of LDH–seaweed composites, as immobilization of bioactives within the LDH matrix may enhance their persistence and efficacy in crop systems. The primary objective of this study was to evaluate the biological activity of composites based on Mg-Al LDHs and alkaline extracts of *S. liebmannii* (LDH-Sargassum) and *U. ohnoi* (LDH-Ulva) using tomato seed germination and mung bean bioassays. To the best of our knowledge, this is the first study to describe the synthesis, physicochemical characterization, and biological activity of LDH-seaweed composites as growth biostimulants during the early stages of plant development. Consequently, there are no prior studies available with which to directly compare our results.

### 4.1 Characterization of composites

The materials were extensively characterized using XRD, TGA–DSC, FT-IR, DLS, SEM, and EDS. These analyses confirmed the successful integration of seaweed-derived compounds onto the LDH structure.

The XRD patterns of the LDH-seaweed composites displayed characteristic reflections corresponding to hydrotalcite-like structures, which was consistent with those reported in previous studies and the database references (Kim et al., 2022; Silva et al., 2022; Ahmad et al., 2024). These patterns confirmed the presence of Mg–Al hydrotalcite crystals in both composites. The diffraction peaks at 10–11° (0 0 3) and 60–62° (1 1 0) (1 1 3), indicated the presence of carbonate as interlayer anions and a uniform distribution of metal cations, respectively (Newman and Jones, 1998; Ahmad et al., 2024). The absence of peak shifts in the (0 0 3) basal reflection relative to pristine LDH suggested that carbonate anions remained with the interlayer space and that seaweed-derived anionic compounds were adsorbed on the outer surface of the LDH particles (Leroux et al., 2004; Jiang et al., 2015). Brucite formation was also considered, particularly in LDH-Ulva, due to the high magnesium oxide content (MgO) in the alkaline extract of *U. ohnoi* (2054.68 ppm) (**Supplementary Table 1**; **Supplementary Figure 2**). Under strongly alkaline extraction conditions (pH 12), this MgO could be transformed into brucite (Pettauer et al., 2024).

The TGA results revealed that approximately 12% of the LDH-seaweed composite dry mass corresponded to the organic matter fraction retained by the LDH matrix, confirming the incorporation of seaweed-derived anionic compounds. The thermal decomposition profile of the composites followed three main stages, similar to those of the pristine LDH (de Castro et al., 2020). The first stage occurred between 25−140 °C and was associated with the removal of weakly adsorbed water (moisture) in the samples (Theiss et al., 2013; de Castro et al., 2020). The second step, from 140 °C up to about 275 °C, was likely related to the removal of interlayer water molecules (Silva et al., 2022; Matsuda et al., 2023) and the decomposition of adsorbed organic compounds (Sharma et al., 2012; Kumar et al., 2021; Hamouda et al., 2022). The third stage, which occurred at temperatures above 300 °C, resulted in the greatest mass loss due to the combustion of organic residues and the dehydroxylation of the structural layers of LDH (de Castro et al., 2020; Silva et al., 2022).

The residual mass at high temperatures represented the ash content, which mainly consisted of Mg–Al mixed oxides formed by structural collapse above 500 °C (Theiss et al., 2013; Guo et al., 2019; Matsuda et al., 2023). Notably, the LDH-seaweed composites had lower residual mass than pristine LDH, indicating reduced thermal stability due to higher organic content (Guo et al., 2019). Differential scanning calorimetry (DSC) further supported these findings, with variations in thermal event temperatures and intensities (150–300 °C) reflecting differences in the composition of the LDH-seaweed composites.

However, TGA-DSC alone could not elucidate the precise nature of these compounds. Therefore, FT-IR spectroscopy provided complementary insights and revealed characteristic vibration bands corresponding to hydroxyl groups, aliphatic C–H bonds, carboxylate groups (C=O), and the glycosidic bridge (C–O–C). Additionally, in LDH-Ulva, absorption bands associated with sulfate groups (S=O and C–S–C) were observed, suggesting the presence of sulfated biomolecules. In contrast, pristine LDH exhibited bands attributable to O–H stretching and metal–oxygen (Mg–O and Al–O) vibrations (Jiang et al., 2015; Zaghloul et al., 2021; Silva et al., 2022; Ahmad et al., 2024).

Given that polysaccharides are major components of the seaweed extracts (Ali et al., 2021), the presence of these functional groups supported their inclusion in the composites. Specifically, alginate is likely present in LDH-Sargassum (Leroux et al., 2004; Nunes et al., 2020), and ulvans are likely present in LDH-Ulva (Kidgell et al., 2019; Macrì et al., 2025). The absence of sulfate-related signals in the FT-IR spectrum of the LDH-Sargassum composite suggests either low content or the absence of fucoidans, which are sulfated polysaccharides typical of brown algae (Pacheco et al., 2021; Macrì et al., 2025). Notably, the high-temperature and alkaline extraction process may have partially degraded these polysaccharides (Sharma et al., 2012; Di Filippo-Herrera et al., 2019), resulting in oligosaccharides detectable by FT-IR. Further analytical techniques are needed to confirm their identities and structures.

The DLS analyses indicated a significant increase in particle size in the aqueous suspensions of the LDH-Sargassum and LDH-Ulva composites compared to pristine LDH. This increase may be attributed to the interaction of seaweed-derived anions with the LDH nanosheets, which likely promoted varying degrees of aggregation among the composites, reflecting the distinct nature of the adsorbed organic compounds, as previously discussed with regard to the FT-IR analysis. Moreover, the turbidity of the suspension decreased over time as a result of particle sedimentation driven by the formation of larger and denser aggregates. This behavior was consistent with previous reports indicating that LDHs synthesized via conventional methods tend to aggregate and precipitate due to their high surface area, particularly at elevated concentrations (Xu et al., 2006; Singha Roy et al., 2022; Ahmad et al., 2024). Secondary aggregates with particle sizes ranging from 1 to 10 µm have been commonly observed under these conditions (Xu et al., 2006). This phenomenon has practical implications for the agricultural application of LDH-seaweed composites.

**Figure 10:**
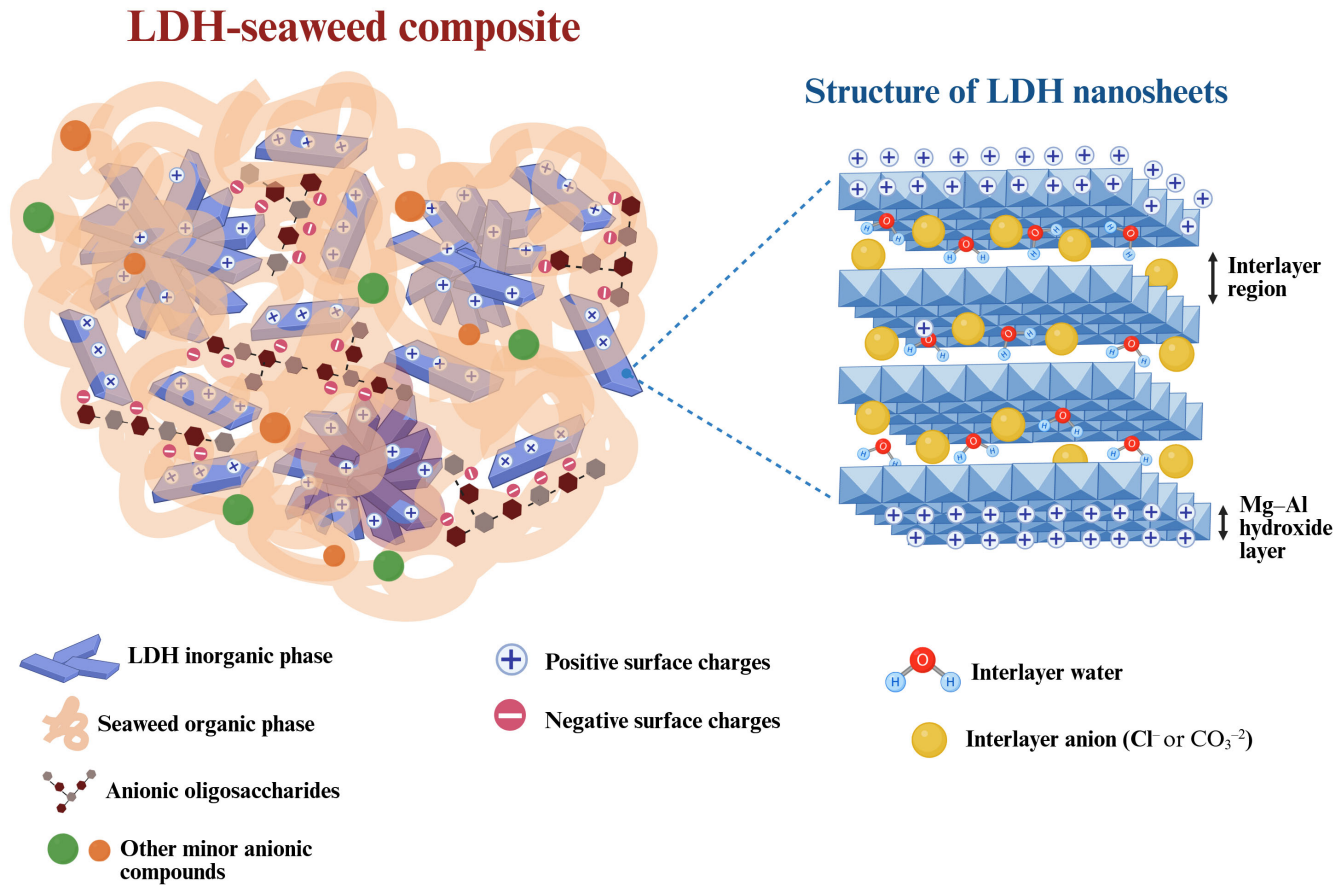
Illustration the proposed architecture of a particle in the layered double hydroxide (LDH)-seaweed composites. Created in https://BioRender.com.

Finally, the EDS analyses demonstrated that the materials predominantly contained oxygen (O), magnesium (Mg), and aluminum (Al). These results support those obtained by XRD and FT-IR, which showed the incorporation of LDH-type structures in the composites. Based on the combined results of the characterization techniques, a structural model of the LDH-seaweed composites is proposed in **Figure 10**.

### 4.2 Effects on tomato seed germination and seedling growth

Seed germination marks the beginning of successful seedling establishment and is crucial for ensuring optimal plant performance in agro-productive settings (Ranal and Santana, 2006; Ribeiro et al., 2024). From a practical point of view, germination bioassays serve as rapid and effective tools to evaluate the potential agricultural applications of novel products (Gupta et al., 2022). Water is a key requirement for seed germination (Hamouda et al., 2022). Thus, the presence and concentration of osmotically active solutes, such as mineral ions or soluble sugars, in the germination medium can modify the osmotic potential, thereby promoting water uptake by seeds and triggering germination (Bewley and Black, 1994; Gupta et al., 2022).

In this study, a similar mechanism may have been at work in seeds treated with the lowest concentrations of the composites, which may have been the result of to the ionic nature of the Mg-Al LDH particles and the presence of anionic organic compounds from the seaweed extracts. The LDH-seaweed composites positively affected tomato seed germination and vigor at the lowest evaluated concentrations. The observed benefits included an increase in the germination percentage and seedling vigor, with the application concentration being the primary factor influencing the germination response to the composites. Moreover, it has been suggested that nanomaterials may enhance seed germination by increasing water permeability (Guo et al., 2022). In this regard, studies have shown that when exposed to low concentrations of carbon-based nanomaterials, seeds of various crop species exhibit greater water uptake and improved germination efficiency. These effects have been linked to the penetration of nanoparticles into seed tissues, leading to the formation of new pores and the activation of aquaporin gene expression in the seed coat (Vithanage et al., 2017).

No adverse effects in seed germination were observed in our study (**Supplementary Table 2**). Although concentrated suspensions were expected to hinder water uptake in seeds, their progressive aggregation in the aqueous suspension may have altered the water potential of the medium or reduced the effective surface area for interactions between the seaweed compounds and seeds, thereby mitigating the anticipated negative impact on germination. Similar behavior has been reported for other nanomaterials in germination assays, where toxicity often does not follow a linear dose-response relationship due to aggregation at higher concentrations (Guo et al., 2022).

In contrast to the findings of this study, previous reports have documented that seed coating or soaking with *Sargassum* and *Ulva* extracts induces dose-dependent effects on germination, with stimulation at low concentrations and inhibition at higher concentrations (Hu et al., 2004; Hernández-Herrera et al., 2014; Ghaderiardakani et al., 2019; Mendoza-Morales et al., 2019; Di Filippo-Herrera et al., 2021). Likewise, high concentrations of metal oxide nanoparticles and other nanomaterials have exhibited toxic effects on seed germination in various crops (Guo et al., 2022; Caser et al., 2024). For example, seed coatings with alginate and Zn-Al LDH composite films intercalated with 1-naphthalenoacetic acid (ZnAl-NAA-LDH) delayed germination in common beans (de Castro et al., 2020). Another study reported that LDH-lactate nanosheets did not affect *Arabidopsis thaliana* seed germination rate within the tested concentration range, whereas the corresponding precursor salts markedly inhibited germination at higher concentrations (Wu et al., 2022).

Significant differences in seedling growth were observed among the LDH-seaweed composite and pristine LDH treatments. This suggests that incorporating anionic compounds from seaweed extracts, which constitute approximately 12% of the total weight of the composites, confers additional bioactive properties. As previously discussed, the primary response we observed was an increase in radicle length at the lowest concentrations, which may have partly resulted from these concentrations accelerating germination. Another possible explanation is that the composite particles adhered to the seed surfaces during the period of seed immersion in the suspensions and subsequently facilitated their direct contact with the radicle post-germination. This can be understood if we consider that the radicle was the first structure to emerge from the seed and that the same concentrations produced opposite effects on hypocotyl length, which was presumably not in contact with the composites. Therefore, we assumed that both LDH-Sargassum and LDH-Ulva contained biologically active compounds that could have stimulated growth upon contact with the radicle.

In this regard, several studies have concluded that primary root growth is one of the main benefits of seaweed extracts when they are applied in low concentrations to various crops, including maize (Hu et al., 2004; Hamouda et al., 2022), tomato (Finnie and van Staden, 1985; Briceño-Domínguez et al., 2014), mung bean (Di Filippo-Herrera et al., 2021), Arabidopsis (Ghaderiardakani et al., 2019), lettuce (Santos et al., 2023), and common bean (Ribeiro et al., 2024) seeds. Thus, seaweed-based products can be recommended as potent natural rooting agents for various agricultural crops, especially when used at appropriate concentrations (Craigie, 2011; Shukla et al., 2025), given that high concentrations may damage plants.

The absence of adverse effects on seed germination and radicle elongation suggests that the LDH-seaweed composites are not phytotoxic, at least not under the experimental conditions evaluated in this study. However, further studies should assess the effects of these compounds on other crops and with different modes of application, as little is known of the potential toxicity mechanisms of LDHs in various organisms, including higher plants (Torbati et al., 2024). Since the toxicity of LDHs is largely determined by the toxicity of their constituent metal ions (Zhang et al., 2025), it is important to highlight that Al³^+^, present in the hydroxylated layers of LDHs, can inhibit seed germination and root growth, as previously reported by Ghaderiardakani et al. (2019) and Wu et al. (2022). However, the presence of Mg²^+^ ions in the LDH nanosheets may partially counteract this adverse effect (Deng et al., 2006). Furthermore, considering the structural stability of LDHs under the conditions used for tomato seed treatments (imbibition in aqueous suspension at neutral pH) (Silva et al., 2022), along with their tendency to aggregate (Xu et al., 2006), the release of Al³^+^ ions into the germination medium is unlikely.

### 4.3 Effects on mung bean rooting

Mung bean has been recognized as a suitable model for identifying root-promoting substances, including seaweed extracts (Stirk and van Staden, 1996; Sharma et al., 2012; Lötze and Hoffman, 2016). Considering the tomato germination results, the rooting activity of the lowest composite concentrations was assessed using a mung bean cutting bioassay. In the present study, both LDH-Sargassum and LDH-Ulva significantly induced adventitious root formation in the cuttings compared to the water-distilled control. Specifically, the LDH-Sargassum treatment resulted in higher rhizogenic activity, as evidenced by an increase in the number of roots and root length, which resulted in a greater accumulation of root biomass. However, treatment with pristine LDH particles did not induce adventitious root formation. In disagreement with these findings, Shlar and Poverenov (2021) reported that a pristine Mg-Al LDH solution did not exert a significant inhibitory effect on the rooting efficiency of mung bean cuttings (number of roots per explant), thereby supporting its low toxicity. Similarly, Wu et al. (2022) demonstrated that nanosheets of Mg-Al LDH-lactate significantly promoted root elongation in Arabidopsis by enhancing polar auxin transport, whereas the corresponding precursor salts [Mg(lactate)_2_ and Al(lactate)_3_] strongly inhibited root growth under the same concentration gradient. Based on these results, the authors suggested that the nanoscale laminar conformation of LDHs modulates the ionic toxicity of the raw materials. The differences observed between those studies and the present work may be attributed to the molar ratio of metal cations (Mg: Al), the concentration range tested, and the exposure duration.

Furthermore, the absence of root-inducing effects in mung bean cuttings treated with pristine LDH suggests that the organic compounds derived from the seaweed extracts were responsible for the observed bioactivity beyond the chemical constituents of the LDH structure itself. After being adsorbed onto the surface of the LDHs, these compounds may have been released in a more controlled and efficient manner in proximity to the cutting in contact with the suspension, which likely enhanced the uptake and physiological effects of the compounds. This mechanism aligns with the results of previous studies indicating that organic constituents, rather than mineral elements, are the primary drivers of the growth-promoting properties of seaweed-based products (Finnie and van Staden, 1985). Moreover, several studies have demonstrated that seaweed extracts elicit auxin-like responses by inducing root formation in mung bean in a manner comparable to synthetic auxins (Sharma et al., 2012; Briceño-Domínguez et al., 2014; Hernández-Herrera et al., 2016).

The results from this bioassay, together with those of the germination tests, provide evidence that the primary biological activity of LDH-seaweed composites is associated with the promotion of root growth. From a practical perspective, these findings are particularly noteworthy, as the development of the root system constitutes a critical morphological characteristic of seedling quality. This attribute directly impacts the transplanting process and, consequently, the successful establishment of seedlings in agricultural settings (Grossnickle and Ivetić, 2022).

Given that LDH-Sargassum and LDH-Ulva showed similar biological activity in all bioassays, we assumed that the compounds responsible for these effects belong to constitutive metabolites, whose presence is not influenced by growth conditions. This assumption is based on the brown alga *S. liebmannii* being collected from wild populations in the intertidal zone, whereas the green alga *U. ohnoi* was obtained from a land-based culture system. Therefore, the observed consistency in bioactivity was unexpected, as it is known to vary according to the species and collection site (Sharma et al., 2012; Hernández-Herrera et al., 2014; Mzibra et al., 2018; Santos et al., 2023). In agreement with our results, Craigie (2011) reported that the chemical composition of the brown alga *Ascophyllum nodosum* was highly consistent across collection sites and seasons during the production of commercial extracts. Furthermore, biological effectiveness tests of these extracts have consistently indicated that root development is promoted in seedlings. These effects have been attributed to an isolated carbohydrate polymer fraction present in these commercial products.

According to the previously discussed results of FT-IR analysis, the biostimulant effect of LDH-seaweed composites on tomato seedling growth and mung bean rooting could be explained by the presence of oligosaccharides derived from algal cell wall polysaccharides adsorbed on LDH surfaces. These biomolecules can act as plant signaling agents by modulating endogenous pathways that regulate growth and development (González et al., 2013; Supriya et al., 2025). For example, oligosaccharides enhance seed germination, seedling development, and root growth by stimulating carbon and nitrogen assimilation, enzymatic activity, basal metabolism, and cell division (Hu et al., 2004; González et al., 2013; Hamouda et al., 2022; Gupta et al., 2022). Considering the above, it can be inferred that the composites may have indirectly triggered metabolic and physiological processes in seeds and seedlings by activating specific signaling pathways. Nevertheless, these hypotheses cannot be substantiated by the experimental results obtained in the current study. Therefore, further bioassays are necessary to enhance our understanding of the mechanisms of action responsible for the observed biological activity.

While both composites demonstrated biostimulant activity during the initial stages of plant growth, LDH-Sargassum induced significantly stronger effects than LDH-Ulva, especially at the concentration of 6.28 mg mL^−1^. These findings may be due to the smaller hydrodynamic size of the LDH-Sargassum particles, which likely increased the surface area available for contact and facilitated greater penetration into plant tissues (Caser et al., 2024). Alternatively, the observed differences may be related to variations in the biochemical composition of the seaweed extracts used as raw materials (**Supplementary Table 1**) and, consequently, of the LDH-seaweed composites, as indicated by the DSC an FT-IR analyses. As previously discussed, oligosaccharides derived from alginates and ulvans may be released by alkaline extraction and subsequently incorporated into LDH sheets during the synthesis of LDH-Sargassum and LDH-Ulva, respectively. Despite being carbohydrates, the bioactivity of cell wall polysaccharides may differ depending on their molecular structure and the conditions of the seaweed habitat (Reyes-Ortega, 2014; Pacheco et al., 2021; Macrì et al., 2025).

## 5 Conclusions

This study presents the first synthesis and physicochemical characterization of Mg-Al LDH composites incorporating alkaline extracts from *Sargassum liebmannii* (LDH-Sargassum) and *Ulva ohnoi* (LDH-Ulva). The LDH matrices effectively adsorbed anionic compounds from the algal extracts, with ~12% organic matter retained on the nanosheets. Compositional differences between the two composites reflected the distinct biochemical profiles of each seaweed.

Biological assays revealed that both composites enhanced seed vigor and radicle elongation in tomato, and stimulated root initiation and growth in mung bean cuttings, outperforming pristine LDH. These effects are likely driven by bioactive oligosaccharides such as alginate and ulvan. The biostimulant activity, combined with the biodegradable and non-phytotoxic nature of the LDH-seaweed composites, underscores their potential as sustainable agricultural inputs. Notably, LDH-Sargassum at 6.28 mg·mL⁻¹ showed strong promise as a natural alternative to synthetic rooting agents.

These findings provide a foundation for the development of LDH-seaweed composites as next-generation phycobiostimulants. Further research should address their performance under field conditions, optimize application rates, and elucidate the molecular mechanisms underlying plant responses. Advancing this platform from proof-of-concept to scalable bioproducts could reduce dependence on synthetic agrochemicals and support more resilient, sustainable agriculture.

## Data Availability

The raw data supporting the conclusions of this article will be made available by the authors, without undue reservation.
