# Supplementary material for: Layered double hydroxide-seaweed composites promote seed germination and seedling growth: a new generation of biostimulants
Source: Front Plant Sci. 2025 Oct 16;16:1681803. doi: 10.3389/fpls.2025.1681803 (PMC12571925; doi:10.3389/fpls.2025.1681803)
Supplement: Supplementary file 1 [file DataSheet1.docx]

Supplementary Material

# Supplementary Tables

**Supplementary Table** **1** Biochemical characterization of alkaline extracts from *Sargassum liebmannii* and *Ulva ohnoi* used as starting materials for the synthesis of LDH-seaweed composites.

| Analysis | Units | *U. ohnoi* | *S. liebmannii* |
| --- | --- | --- | --- |
| Organic matter | % | 1.54 | 1.07 |
| Carbon | % | 0.89 | 0.62 |
| Nitrogen | % | 1.05 | 0.64 |
| Carbon/nitrogen ratio |  | 0.85 | 0.97 |
| Phosphates (P-PO_4_^–^) | ppm | 64.00 | 56.00 |
| Sulfates (SO_4_^–2^) | ppm | 1060.00 | 900.00 |
| Sulfur sulphates (S-SO_4_^–2^) | ppm | 353.33 | 300.00 |
| Magnesium oxide (MgO) | ppm | 2054.68 | 205.47 |
| Magnesium (Mg^+2^) | ppm | 1240.00 | 124.00 |
| Total phenols | mg GAE⋅g^−^¹ DM | 144.23 | 126.48 |
| Total flavonoids | mg QE⋅g^−^¹ DM | 12.41 | 6.08 |
| Reducing sugars | mg DE⋅g^−^¹ DM | 44.03 | 35.28 |

GAE, gallic acid equivalents; QE, quercetin equivalents; DE, dextrose equivalents; DM, dry matter

**Supplementary Table** **2.** Average values of variables analyzed in tomato germination tests (*n* = 3). The 0 mg·mL^−1^ concentration (distilled water) was used as the control group. GP, germination percentage; VI-1, Vigor index 1; VI-2, Vigor index 2; RL, radicle length; HL, hypocotyl length; SL, seedling length; DW, dry weight.

|  |  | **Concentration (mg**·**mL^−1^)** | | | | | | |
| --- | --- | --- | --- | --- | --- | --- | --- | --- |
| **Variable** | **Treatment** | 0 | 1.57 | 3.14 | 6.28 | 12.56 | 25.12 | 50.25 |
| **Germination** | | | | | | | | |
| PG (%) | LDH | 85.86 | 91.92 | 91.92 | 92.93 | 84.85 | 89.90 | 92.93 |
|  | LDH-Sargassum | – | 95.96 | 91.92 | 94.95 | 88.89 | 91.92 | 85.86 |
|  | LDH-Ulva | – | 95.96 | 91.92 | 89.90 | 90.91 | 84.85 | 91.92 |
| VI-1 | LDH | 1389.81 | 1501.28 | 1469.55 | 1395.33 | 1336.19 | 1406.60 | 1541.90 |
|  | LDH-Sargassum | – | 1646.50 | 1559.24 | 1674.16 | 1501.03 | 1619.38 | 1429.75 |
|  | LDH-Ulva | – | 1607.82 | 1483.60 | 1629.69 | 1580.07 | 1496.94 | 1617.62 |
| VI-2 | LDH | 38.62 | 34.93 | 35.33 | 37.06 | 34.67 | 33.77 | 36.17 |
|  | LDH-Sargassum | – | 45.74 | 42.80 | 47.02 | 35.13 | 50.15 | 34.20 |
|  | LDH-Ulva | – | 40.93 | 35.99 | 40.62 | 32.91 | 34.73 | 35.16 |
| **Seedling growth** | | | | | | | | |
| HL (cm) | LDH | 9.24 | 9.38 | 9.05 | 9.22 | 8.84 | 8.36 | 8.74 |
|  | LDH-Sargassum | – | 6.93 | 6.97 | 7.65 | 8.31 | 8.64 | 8.42 |
|  | LDH-Ulva | – | 7.56 | 7.54 | 8.14 | 8.72 | 8.58 | 8.55 |
| RL (cm) | LDH | 6.95 | 6.95 | 6.93 | 5.78 | 6.92 | 7.26 | 7.86 |
|  | LDH-Sargassum | – | 10.23 | 10.65 | 9.99 | 8.56 | 8.96 | 8.22 |
|  | LDH-Ulva | – | 9.23 | 9.20 | 9.87 | 8.67 | 9.06 | 9.19 |
| SL (cm) | LDH | 16.19 | 16.34 | 15.99 | 15.00 | 15.76 | 15.62 | 16.60 |
|  | LDH-Sargassum | – | 17.16 | 16.96 | 18.00 | 16.87 | 17.60 | 16.64 |
|  | LDH-Ulva | – | 16.75 | 16.13 | 17.63 | 17.38 | 17.65 | 17.73 |
| DW (mg) | LDH | 15.5 | 15.10 | 13.50 | 14.00 | 16.00 | 13.40 | 13.40 |
|  | LDH-Sargassum | – | 15.40 | 15.90 | 17.40 | 15.00 | 16.40 | 14.80 |
|  | LDH-Ulva | – | 15.60 | 14.70 | 15.00 | 14.80 | 16.60 | 13.80 |

**Supplementary Table** **3.** Results of the post-hoc tests of the two-way crossed PERMANOVA of the Concentration × Treatment interactions for tomato germination parameters. The 0 mg·mL^−1^ concentration (distilled water) was used as the control group. Bold numbers indicate significant differences (*P* ≤ 0.05) based on Monte Carlo test.

| Term 'Interaction' for pairs of levels of factor 'Concentration' | | | | | | |
| --- | --- | --- | --- | --- | --- | --- |
| **Groups** | **Treatment** | | | | | |
|  | LDH | | LDH-Sargassum | | LDH-Ulva | |
|  | t | *P*(MC) | t | *P*(MC) | t | *P*(MC) |
| 0 *vs.* 1.57 mg mL^−1^ | 1.969 | 0.105 | 3.262 | **0.017** | 3.441 | **0.017** |
| 0 *vs.* 3.14 mg mL^−1^ | 1.662 | 0.126 | 2.077 | 0.080 | 1.693 | 0.126 |
| 0 *vs.* 6.28 mg mL^−1^ | 1.324 | 0.231 | 4.304 | **0.008** | 2.513 | **0.044** |
| 0 *vs.* 12.56 mg mL^−1^ | 0.982 | 0.417 | 1.075 | 0.338 | 2.130 | 0.052 |
| 0 *vs.* 25.12 mg mL^−1^ | 0.930 | 0.423 | 1.803 | 0.128 | 1.309 | 0.247 |
| 0 *vs.* 50.25 mg mL^−1^ | 2.495 | **0.043** | 0.785 | 0.543 | 3.186 | **0.019** |
| 1.57 *vs.* 3.14 mg mL^−1^ | 0.307 | 0.945 | 1.214 | 0.275 | 1.940 | 0.078 |
| 1.57 *vs.* 6.28 mg mL^−1^ | 0.846 | 0.513 | 0.459 | 0.807 | 1.999 | 0.072 |
| 1.57 *vs.* 12.56 mg mL^−1^ | 2.803 | **0.028** | 2.183 | 0.085 | 2.009 | 0.089 |
| 1.57 *vs.* 25.12 mg mL^−1^ | 0.732 | 0.536 | 0.699 | 0.561 | 4.052 | **0.011** |
| 1.57 *vs.* 50.25 mg mL^−1^ | 0.632 | 0.707 | 3.231 | **0.021** | 2.244 | 0.059 |
| 3.14 *vs.* 6.28 mg mL^−1^ | 0.592 | 0.688 | 1.673 | 0.141 | 1.745 | 0.095 |
| 3.14 *vs.* 12.56 mg mL^−1^ | 2.312 | **0.042** | 1.275 | 0.255 | 0.980 | 0.417 |
| 3.14 *vs.* 25.12 mg mL^−1^ | 0.560 | 0.685 | 0.790 | 0.519 | 1.917 | 0.069 |
| 3.14 *vs.* 50.25 mg mL^−1^ | 0.802 | 0.537 | 2.135 | 0.071 | 1.612 | 0.149 |
| 6.28 *vs.* 12.56 mg mL^−1^ | 1.726 | 0.123 | 2.688 | **0.049** | 1.547 | 0.154 |
| 6.28 *vs.* 25.12 mg mL^−1^ | 0.586 | 0.661 | 0.614 | 0.635 | 2.601 | **0.046** |
| 6.28 *vs.* 50.25 mg mL^−1^ | 1.108 | 0.330 | 4.140 | **0.011** | 1.760 | 0.102 |
| 12.56 *vs.* 25.12 mg mL^−1^ | 1.041 | 0.356 | 1.544 | 0.191 | 1.728 | 0.111 |
| 12.56 *vs.* 50.25 mg mL^−1^ | 3.741 | **0.010** | 0.732 | 0.521 | 0.737 | 0.554 |
| 25.12 *vs.* 50.25 mg mL^−1^ | 1.124 | 0.328 | 1.976 | 0.103 | 3.136 | **0.024** |
| Term 'Interaction' for pairs of levels of factor 'Treatment' | | | | | | |
| **Groups** | **Concentration** | | | | | |
|  | 1.57 mg mL^−1^ | | 3.14 mg mL^−1^ | | 6.28 mg mL^−1^ | |
|  | t | *P*(MC) | t | *P*(MC) | t | *P*(MC) |
| LDH *vs.* LDH-Sargassum | 2.531 | **0.041** | 1.484 | 0.169 | 2.692 | **0.036** |
| LDH *vs.* LDH-Ulva | 2.176 | **0.050** | 0.169 | 0.987 | 1.847 | 0.087 |
| LDH-Sargassum *vs.* LDH-Ulva | 0.979 | 0.385 | 1.339 | 0.225 | 2.668 | **0.035** |
| **Groups** | **Concentration** | | | | | |
|  | 12.56 mg mL^−1^ | | 25.12 mg mL^−1^ | | 50.25 mg mL^−1^ | |
|  | t | *P*(MC) | t | *P*(MC) | t | *P*(MC) |
| LDH *vs.* LDH-Sargassum | 1.498 | 0.183 | 1.737 | 0.137 | 2.076 | 0.070 |
| LDH *vs.* LDH-Ulva | 2.747 | **0.019** | 1.103 | 0.337 | 1.363 | 0.219 |
| LDH-Sargassum *vs.* LDH-Ulva | 0.716 | 0.576 | 1.989 | 0.116 | 2.516 | **0.044** |

**Supplementary Table** **4.** Results of the post-hoc tests of the two-way crossed PERMANOVA of the Concentration × Treatment interactions for tomato seedling growth parameters. The 0 mg·mL^−1^ concentration (distilled water) was used as the control group. Bold numbers indicate significant differences (*P* ≤ 0.05) based on Monte Carlo test.

| Term 'Interaction' for pairs of levels of factor 'Concentration' | | | | | | |
| --- | --- | --- | --- | --- | --- | --- |
| **Groups** | **Treatment** | | | | | |
|  | LDH | | LDH-Sargassum | | LDH-Ulva | |
|  | t | *P*(MC) | t | *P*(MC) | t | *P*(MC) |
| 0 *vs.* 1.57 mg mL^−1^ | 0.647 | 0.645 | 6.111 | **0.001** | 3.514 | **0.006** |
| 0 *vs.* 3.14 mg mL^−1^ | 1.304 | 0.239 | 3.893 | **0.005** | 2.996 | **0.013** |
| 0 *vs.* 6.28 mg mL^−1^ | 2.970 | **0.013** | 6.169 | **0.001** | 5.141 | **0.002** |
| 0 *vs.* 12.56 mg mL^−1^ | 0.670 | 0.654 | 2.858 | **0.015** | 2.336 | **0.027** |
| 0 *vs.* 25.12 mg mL^−1^ | 2.667 | **0.020** | 2.847 | **0.019** | 4.510 | **0.002** |
| 0 *vs.* 50.25 mg mL^−1^ | 2.114 | **0.048** | 1.855 | 0.065 | 3.459 | **0.008** |
| 1.57 *vs.* 3.14 mg mL^−1^ | 1.147 | 0.315 | 0.560 | 0.723 | 0.948 | 0.427 |
| 1.57 *vs.* 6.28 mg mL^−1^ | 3.344 | **0.013** | 3.658 | **0.008** | 2.319 | **0.045** |
| 1.57 *vs.* 12.56 mg mL^−1^ | 1.038 | 0.380 | 3.833 | **0.003** | 1.964 | 0.079 |
| 1.57 *vs.* 25.12 mg mL^−1^ | 2.828 | **0.015** | 3.374 | **0.010** | 2.798 | **0.022** |
| 1.57 *vs.* 50.25 mg mL^−1^ | 2.011 | 0.052 | 3.167 | **0.009** | 2.423 | **0.049** |
| 3.14 *vs.* 6.28 mg mL^−1^ | 1.204 | 0.289 | 1.713 | 0.108 | 2.471 | **0.049** |
| 3.14 *vs.* 12.56 mg mL^−1^ | 1.284 | 0.246 | 2.402 | **0.027** | 1.949 | 0.082 |
| 3.14 *vs.* 25.12 mg mL^−1^ | 0.814 | 0.536 | 2.319 | **0.030** | 3.111 | **0.025** |
| 3.14 *vs.* 50.25 mg mL^−1^ | 0.772 | 0.587 | 2.393 | **0.026** | 2.190 | 0.051 |
| 6.28 *vs.* 12.56 mg mL^−1^ | 1.761 | 0.128 | 4.647 | **0.002** | 1.721 | 0.089 |
| 6.28 *vs.* 25.12 mg mL^−1^ | 2.419 | **0.027** | 2.303 | **0.031** | 3.671 | **0.007** |
| 6.28 *vs.* 50.25 mg mL^−1^ | 2.826 | **0.018** | 3.673 | **0.010** | 1.611 | 0.133 |
| 12.56 *vs.* 25.12 mg mL^−1^ | 1.752 | 0.114 | 1.853 | 0.100 | 2.126 | 0.053 |
| 12.56 *vs.* 50.25 mg mL^−1^ | 1.760 | 0.103 | 0.517 | 0.771 | 0.976 | 0.433 |
| 25.12 *vs.* 50.25 mg mL^−1^ | 1.272 | 0.260 | 1.825 | 0.114 | 3.265 | **0.023** |
| Term 'Interaction' for pairs of levels of factor 'Treatment' | | | | | | |
| **Groups** | **Concentration** | | | | | |
|  | 1.57 mg mL^−1^ | | 3.14 mg mL^−1^ | | 6.28 mg mL^−1^ | |
|  | t | *P*(MC) | t | *P*(MC) | t | *P*(MC) |
| LDH *vs.* LDH-Sargassum | 7.301 | **<0.001** | 3.063 | **0.008** | 10.415 | **<0.001** |
| LDH *vs.* LDH-Ulva | 4.069 | **0.003** | 2.024 | **0.042** | 8.510 | **<0.001** |
| LDH-Sargassum *vs.* LDH-Ulva | 1.630 | 0.103 | 1.484 | 0.172 | 4.113 | **0.003** |
| **Groups** | **Concentration** | | | | | |
|  | 12.56 mg mL^−1^ | | 25.12 mg mL^−1^ | | 50.25 mg mL^−1^ | |
|  | t | *P*(MC) | t | *P*(MC) | t | *P*(MC) |
| LDH *vs.* LDH-Sargassum | 1.711 | 0.108 | 3.840 | **0.009** | 1.152 | 0.319 |
| LDH *vs.* LDH-Ulva | 1.853 | 0.066 | 5.543 | **0.002** | 1.640 | 0.121 |
| LDH-Sargassum *vs.* LDH-Ulva | 0.984 | 0.415 | 0.297 | 0.913 | 1.664 | 0.120 |

**Supplementary Table** **5.** Results of the post-hoc tests of the two-way crossed PERMANOVA of the Concentration × Treatment interactions for the mung bean rooting variables. Bold numbers indicate significant differences (*P* ≤ 0.05) based on Monte Carlo test.

| Term 'Interaction' for pairs of levels of factor 'Concentration' | | | | |
| --- | --- | --- | --- | --- |
| **Groups** | **Treatment** | | | |
|  | LDH-Sargassum | | LDH-Ulva | |
|  | t | *P*(MC) | t | *P*(MC) |
| 0 *vs.* 1.57 mg mL^−1^ | 14.994 | **<0.001** | 2.614 | **0.007** |
| 0 *vs.* 3.14 mg mL^−1^ | 17.078 | **<0.001** | 3.798 | **<0.001** |
| 0 *vs.* 6.28 mg mL^−1^ | 19.052 | **<0.001** | 9.598 | **<0.001** |
| 1.57 *vs.* 3.14 mg mL^−1^ | 3.215 | **0.001** | 5.421 | **<0.001** |
| 1.57 *vs.* 6.28 mg mL^−1^ | 2.911 | **0.005** | 10.392 | **<0.001** |
| 3.14 *vs.* 6.28 mg mL^−1^ | 1.922 | **0.038** | 5.767 | **<0.001** |
| Term 'Interaction' for pairs of levels of factor 'Treatment' | | | | |
| **Groups** | Concentration | | | |
|  | 1.57 mg mL^−1^ | | 3.14 mg mL^−1^ | |
|  | t | *P*(MC) | t | *P*(MC) |
| LDH-Sargassum *vs.* LDH-Ulva | 14.484 | **<0.001** | 14.995 | **<0.001** |
| **Groups** | Concentration | | | |
|  | 6.28 mg mL^−1^ | |  |  |
|  | t | *P*(MC) |  |  |
| LDH-Sargassum *vs.* LDH-Ulva | 13.179 | **<0.001** |  |  |

# Supplementary Figures


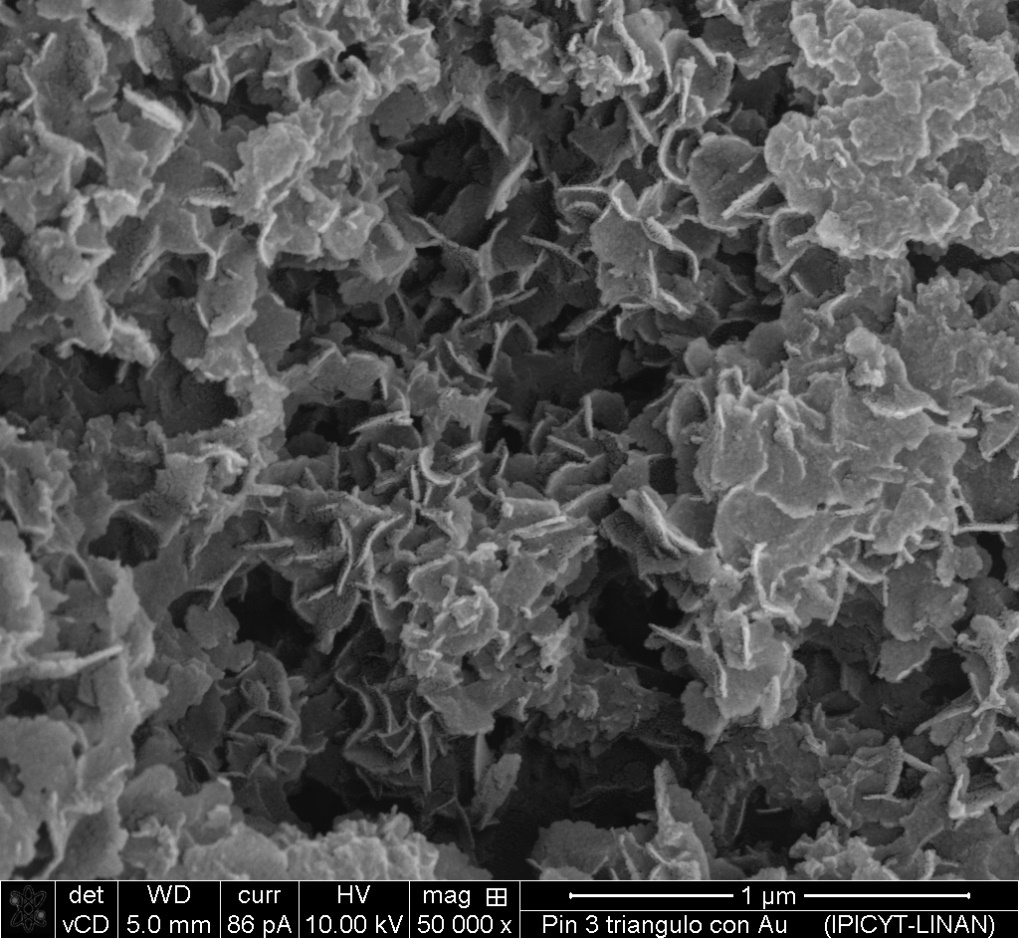


**Supplementary Figure** **1** SEM image of the Mg–Al layered double hydroxides (LDH). The sample was coated with gold (Au) to facilitate the observation of the "desert rose" type arrangement of the LDH nanolayers. The micrograph was obtained using a Helios Nanolab 600 at a magnification of 50,000×.


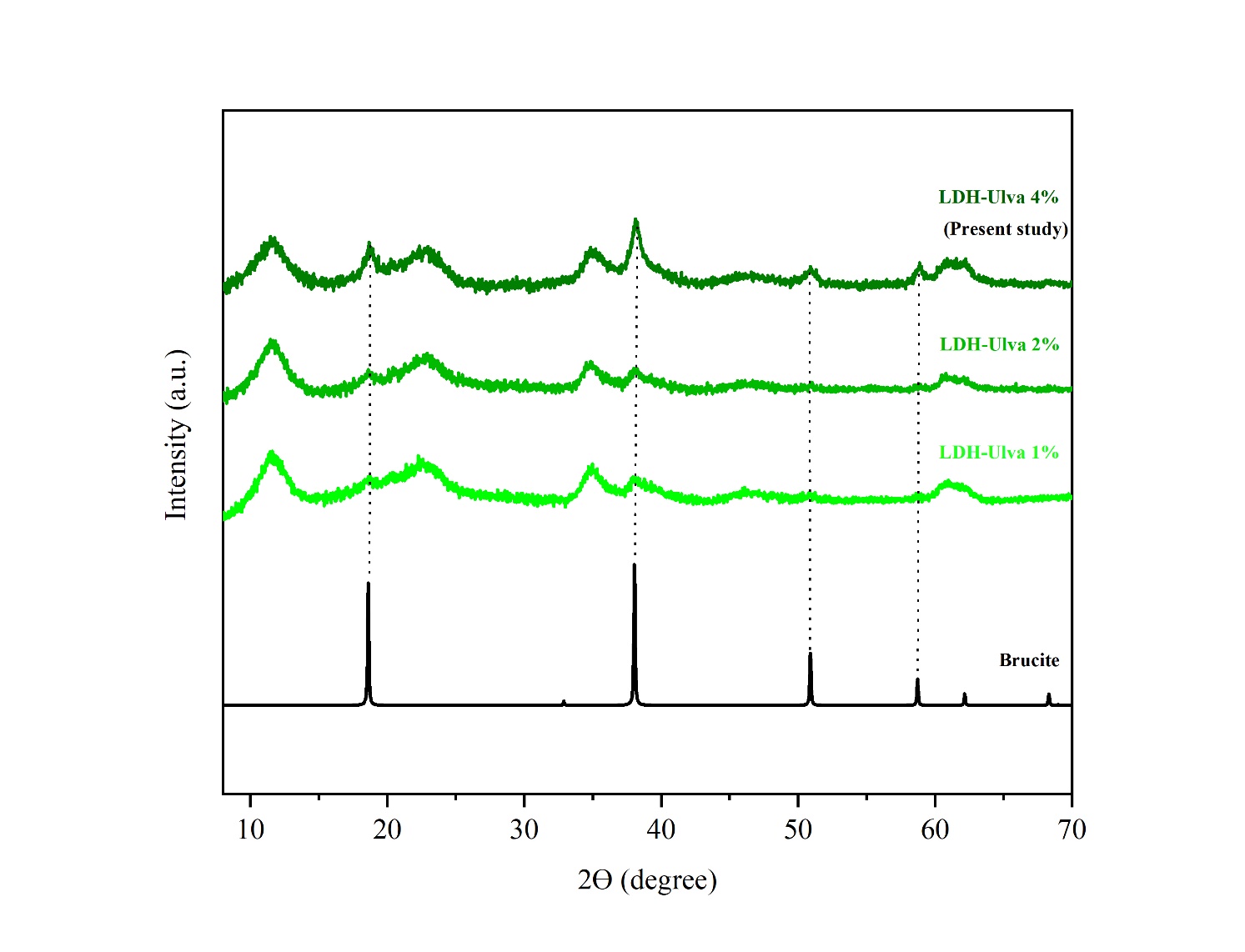


**Supplementary Figure** **2** X-ray diffraction patterns of LDH-Ulva composites synthesized from alkaline extracts of *Ulva ohnoi* at concentrations of 1%, 2%, and 4%. The intensity of the characteristic brucite [Mg(OH_2_)] signals progressively decreases with increasing extract dilution.
